# A cardiac-related, promoter-proximal, regulatory element shapes chromatin and JAG1 transcription

**DOI:** 10.26508/lsa.202503597

**Published:** 2026-08-03

**Authors:** Quannan Zhuang, Feng Wang, Huinan Zhang, Qi Zhang, Yuan Gao, Zhiyu Feng, Han Gao, Siyu Sun, Siyi Lin, Shuolin Li, Quming Zhao, Xianghui Huang, Qiang Li, Wei Sheng, Guoying Huang

**Affiliations:** 1 Shanghai Key Laboratory of Birth Defects and Pediatric Heart Center, Children’s Hospital of Fudan University , Shanghai, China; 2 Department of Respiratory Medicine, Children’s Hospital of Fudan University , Shanghai, China; 3 Department of Pediatrics, Peking Union Medical College Hospital, Chinese Academy of Medical Sciences and Peking Union Medical College, Beijing, China; 4 Fujian Key Laboratory of Neonatal Diseases, Xiamen Children’s Hospital , Fujian, China; 5 Translational Medical Center for Development and Disease, NHC Key Laboratory of Neonatal Diseases, Children’s Hospital of Fudan University , Shanghai, China

## Abstract

The *Jagged1* (*JAG1*) gene is essential for cardiac development, yet its tissue-specific transcriptional regulation remains poorly understood. In this study we used an integrative screening approach to identify 19 candidate enhancers within the ±100 kb region flanking the *JAG1* locus, among which R7 exhibited the highest activity in dual-luciferase assays. CRISPR/Cas9-mediated deletion of R7 in AC16 cells significantly reduced *JAG1* expression, decreased proliferative and migratory capacities, and increased apoptosis. Mechanistically, R7 deletion altered local chromatin contacts and reduced accessibility at CTCF-bound regions near the *JAG1* promoter, accompanied by decreased H3K27ac, H3K4me3, RNA polymerase II, and SRF occupancy. These findings identify R7 as a cardiac-associated promoter-proximal regulatory element with enhancer-like activity that contributes to local chromatin organization and transcriptional activity at the *JAG1* locus.

## Abstract (precis)

This study identifies R7 as a promoter-proximal element that supports *JAG1* transcription by coordinating local chromatin contacts and regulatory features.

## Introduction

*Jagged1* (*JAG1*), as one of the ligands in the highly conserved Notch signaling pathway, plays an essential role in mammalian development, particularly in cardiogenesis, and is closely associated with cardiac development and disease (MacGrogan et al, 2018). During murine cardiac development, myocardial *JAG1* activates endocardial Notch1 signaling to promote myocardial structural organization and maturation, also inhibiting mesenchymal cell proliferation to ensure proper endocardial cushion fusion (D'Amato et al, 2016; MacGrogan et al, 2016). Both syndromic and isolated congenital heart disease (CHD) have been linked to mutations in *JAG1* (Zaidi & Brueckner, 2017). Alagille syndrome is primarily caused by pathogenic variants in *JAG1*, identified in 94.3% of clinically diagnosed patients, and cardiac anomalies are reported in approximately 91% of patients with ALGS (Gilbert et al, 2019; Vandriel et al, 2023). Multiple studies have identified missense mutations in *JAG1* among patients with tetralogy of Fallot (TOF) and demonstrated a strong association between *JAG1* mutations and TOF pathogenesis (Eldadah et al, 2001; Morgenthau & Frishman, 2018). Although the coding function of *JAG1* in cardiac development has been extensively studied, how its tissue-associated transcription is regulated remains incompletely characterized.

Noncoding cis-regulatory elements, including enhancers, play essential roles in the precise regulation of gene expression during human development and homeostasis (Gasperini et al, 2020). Enhancer-associated regulatory regions can influence the accessibility of genomic regions to transcription factors (TFs) and regulatory proteins and then mediate structural interactions between promoters and distal regulatory elements to control target-gene expression (Dixon et al, 2015). Dynamic changes in the enhancer landscape underlie the precise temporal and spatial morphological events during cardiac development by regulating gene expression programs (Anene-Nzelu et al, 2022). Previous studies have demonstrated that single nucleotide polymorphisms (SNPs) in enhancer loci may disrupt the expression of target genes by altering enhancer activity, thereby contributing to disease pathogenesis (Wamstad et al, 2014). The SNP (rs6801957) in the *SCN10A* enhancer disrupts TBX3/TBX5 binding, reducing its cardiac enhancer activity and altering human cardiac conduction patterns (van den Boogaard et al, 2012). In the CHD patient cohort, noncoding de novo variants (DNVs) contribute to disease through both transcriptional and post-transcriptional regulation, with the proportion of predicted functional DNVs being at least comparable to that of coding DNVs (Richter et al, 2020). To date, the cis-regulatory landscape controlling *JAG1* expression in cardiac contexts remains incompletely characterized, leaving gaps in our understanding of its tissue-associated transcriptional regulation. Given that the full spectrum of *JAG1* regulatory regions has yet to be comprehensively identified, systematic discovery and functional characterization of heart-associated regulatory elements are essential for elucidating their roles in cardiac gene regulation and pathogenesis of CHD.

Advances in high-throughput sequencing and epigenomic profiling strategies have accelerated the identification of candidate cis-regulatory elements. Chromatin immunoprecipitation sequencing (ChIP-seq) provides an effective genome-wide approach for identifying candidate regulatory regions by mapping enhancer-associated histone modifications, such as H3K27ac, transcriptional regulators, and mediator complexes (Heintzman et al, 2009; ENCODE Project Consortium et al, 2020).

However, assigning regulatory function and target-gene specificity to candidate elements across different cell types and developmental stages remains challenging (Galouzis & Furlong, 2022; Nair et al, 2022). In reporter-based functional screening, massively parallel reporter assays (MPRAs) and high-throughput approaches can be performed in induced pluripotent stem cell-derived cardiomyocytes (iPSC-CMs) to evaluate cardiac regulatory activity (Xiao et al, 2024). Integrating heart-enriched transcription factor occupancy, chromatin accessibility, and enhancer-associated histone modifications enables more precise prioritization of cardiac regulatory candidates (Akerberg et al, 2019). Therefore, an efficient strategy is needed to screen and functionally characterize heart-associated regulatory elements at the *JAG1* locus.

In this study, we used the R-panel method to screen candidate heart-associated enhancers within a ±100 kb region surrounding the *JAG1* locus. We focused on R7, a promoter-proximal candidate regulatory region showing the strongest reporter activity, and examined its contribution to *JAG1* transcription, AC16 cell phenotypes, and local chromatin regulation.

## Results

### Integrative screening of heart-associated enhancer candidates near *JAG1* identifies R7 as a promoter-proximal regulatory element with enhancer-like activity

To explore heart-associated enhancer candidates surrounding the *JAG1* locus, we used a previously established tissue-specific enhancer prediction pipeline, R-panel (Wang et al, 2022). Within a ±100 kb window centered on the *JAG1* locus, a total of 19 candidate enhancer fragments were identified. These fragments (29–188 bp; average: 92 bp) were distributed from distal upstream regions (>80 kb) to intragenic segments of *JAG1* (Table 1). Based on their genomic localization relative to *JAG1*, fragments with an inter-peak distance <1,000 bp were merged into candidate enhancer clusters to facilitate functional screening, resulting in 10 enhancer regions (R1–R10) for subsequent analysis (Table 1).

**Table 1.** Information on candidate heart-associated enhancers.

Name	Number	Genomic position	Length (bp)	Distance to gene
R1	E1	chr20:10532341-10532692	352	85640
R2	E2	chr20:10539367-10539608	242	78724
R3	E3	chr20:10601567-10601733	167	16599
​	E4	chr20:10602140-10602218	79	16114
R4	E5	chr20:10617037-10617136	100	1196
​	E6	chr20:10617277-10617341	65	991
​	E7	chr20:10617376-10617433	58	899
​	E8	chr20:10617562-10617687	126	645
​	E9	chr20:10617778-10617807	30	525
R5	E10	chr20:10652456-10652643	188	0
R6	E11	chr20:10654361-10654389	29	0
​	E12	chr20:10654396-10654493	98	0
R7	E13	chr20:10654947-10655061	115	253
​	E14	chr20:10655073-10655084	12	379
​	E15	chr20:10655396-10655457	62	702
R8	E16	chr20:10709489-10709767	279	54795
R9	E17	chr20:10722532-10722697	166	67838
​	E18	chr20:10722795-10723107	313	68101
R10	E19	chr20:10742015-10742049	35	87321

Candidate fragments were identified within the ±100 kb region surrounding the *JAG1* locus using the R-panel strategy. Nearby fragments separated by <1,000 bp were grouped into R1–R10 for luciferase screening. Genomic coordinates are based on the human hg19 genome assembly. Distance to gene indicates the distance to *JAG1*; fragments located within *JAG1* are shown as 0.

**Figure 1.**
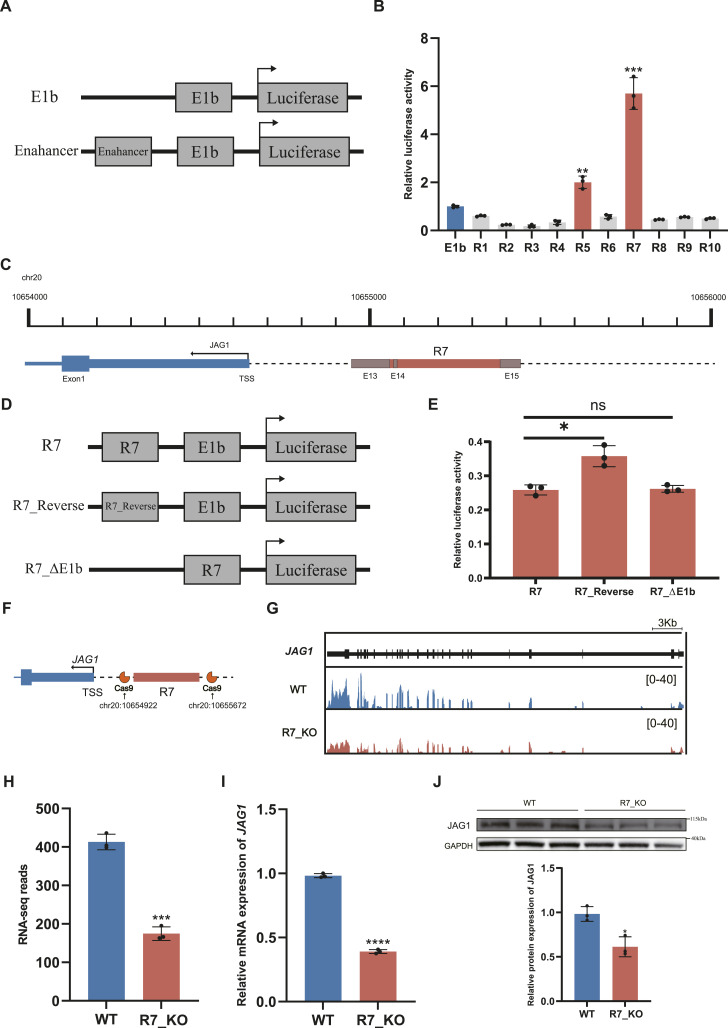
Identification of R7 as a promoter-proximal regulatory element with enhancer-like activity at the *JAG1* locus. **(A)** Schematic of the E1b minimal promoter luciferase reporter system. Candidate regions were cloned upstream of E1b. **(B)** Luciferase activity of candidate regions R1–R10 in AC16 cells. R5 and R7 showed significantly higher activity than the E1b control, with R7 showing the strongest activity. **(C)** Genomic location of R7 relative to the *JAG1* transcription start site (TSS). R7 is located 253 bp upstream of the *JAG1* TSS. **(D)** Schematic of R7 reporter constructs, including forward R7 upstream of E1b (R7), reverse-orientation R7 upstream of E1b (R7_Reverse), and R7 upstream of luciferase without the E1b minimal promoter (R7_ΔE1b). **(E)** Luciferase activity of the R7 constructs shown in (D). R7_Reverse retained reporter activity, whereas R7_ΔE1b showed activity comparable with the forward R7 construct. **(F)** Schematic of CRISPR/Cas9-mediated R7 deletion in AC16 cells. **(G)** RNA-seq read coverage across the *JAG1* locus in WT and R7_KO cells. **(H)** RNA-seq quantification of *JAG1* transcript abundance. **(I)** RT-qPCR analysis of *JAG1* mRNA expression. **(J)** Western blot analysis and quantification of JAG1 protein expression. Data are shown as mean ± SD. Statistical significance was determined by one-way ANOVA with Dunnett’s test for panels B and E, and by two-tailed unpaired *t* test for panels H, I, J. *P < 0.05, **P < 0.01, ***P < 0.001, ****P < 0.0001; ns, not significant.

To evaluate enhancer-associated reporter activity, all candidate regions were cloned upstream of a minimal E1b promoter and tested in AC16 cells using dual-luciferase assays (Fig 1A). R5 and R7 showed significantly higher activity than the baseline pGL3-E1b vector (Fig 1B). Genomic mapping revealed that R7 is located 253 bp upstream of the *JAG1* transcription start site (TSS), placing it in close proximity to the promoter region (Fig 1C). Thus, although R7 was identified through an enhancer-associated screening strategy and showed the strongest reporter activity, its close proximity to the *JAG1* TSS prompted further evaluation using additional reporter configurations. We tested three R7 reporter constructs: R7 cloned upstream of the E1b minimal promoter in the forward orientation (R7), R7 cloned upstream of E1b in the opposite orientation to test orientation-dependent reporter activity (R7_Reverse), and R7 cloned directly upstream of the luciferase gene without the E1b minimal promoter (R7_ΔE1b) (Fig 1D). The forward R7-E1b construct showed reporter activity, and this activity was maintained when R7 was inserted in the reverse orientation, supporting enhancer-like reporter activity. In addition, R7_ΔE1b retained activity comparable with the forward R7-E1b construct, suggesting promoter-like reporter activity in this assay (Fig 1E). These findings indicate that R7 displays enhancer-like activity together with promoter-like reporter activity in this assay, supporting its designation as a promoter-proximal regulatory element.

To investigate the endogenous regulatory impact of R7 on *JAG1* expression, we generated an R7-deletion AC16 cell line (R7_KO) using CRISPR/Cas9-mediated genome editing (Fig 1F). RNA-seq read coverage and quantification across the *JAG1* locus both revealed a substantial decrease in *JAG1* transcript abundance in R7_KO cells compared with wild-type (WT) cells (Fig 1G and H). Consistently, *JAG1* expression was significantly down-regulated at both the mRNA and protein levels in R7_KO cells, as shown by reverse-transcription quantitative PCR (RT-qPCR) and Western blot analyses (Fig 1I and J). Together, these results demonstrate that R7 is required for maintaining *JAG1* transcription in AC16 cells and support its role as a promoter-proximal regulatory element at the *JAG1* locus.

### R7 deletion causes cell-cycle, viability, and apoptosis defects that are partially alleviated by restoration of *JAG1* expression

**Figure 2.**
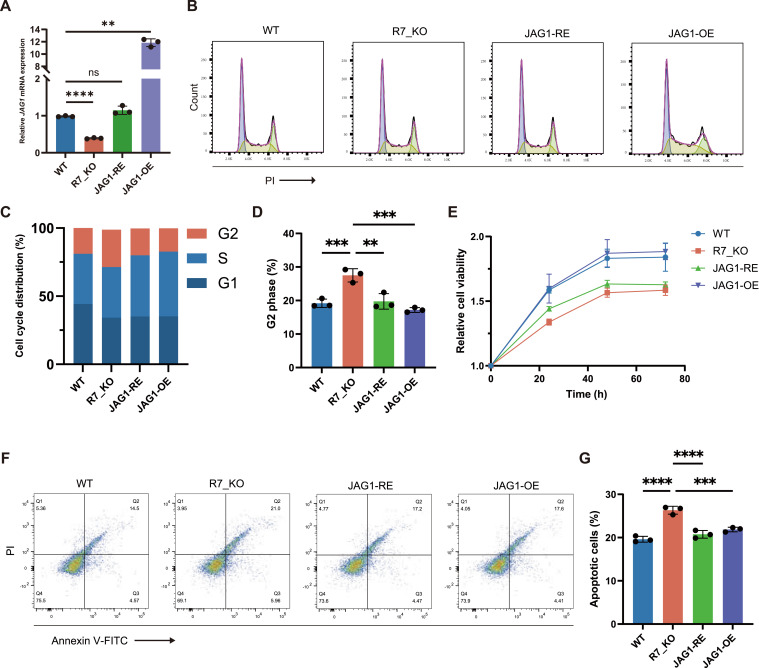
R7 deletion affects AC16 cell-cycle progression, cell viability, and apoptosis, with partial alleviation by *JAG1* restoration or overexpression. **(A)** RT-qPCR analysis of *JAG1* mRNA expression in WT, R7_KO, JAG1-RE, and JAG1-OE cells. JAG1-RE indicates restoration of *JAG1* expression to near-WT levels, whereas JAG1-OE indicates higher *JAG1* overexpression in R7_KO cells. **(B)** Representative PI-staining flow-cytometry profiles for cell-cycle analysis. **(C)** Quantification of cell-cycle phase distribution. **(D)** Quantification of G2/M-phase cells. R7 deletion increased the G2/M population, which was reduced by *JAG1* restoration or overexpression. **(E)** CCK-8 assay showing relative cell viability over time. **(F)** Representative Annexin V-FITC/PI flow-cytometry plots for apoptosis analysis. **(G)** Quantification of apoptotic cells. Data are shown as mean ± SD. Statistical significance was determined by a two-tailed unpaired *t* test for the indicated comparisons. **P < 0.01, ***P < 0.001, ****P < 0.0001; ns, not significant.

**Figure S1.**
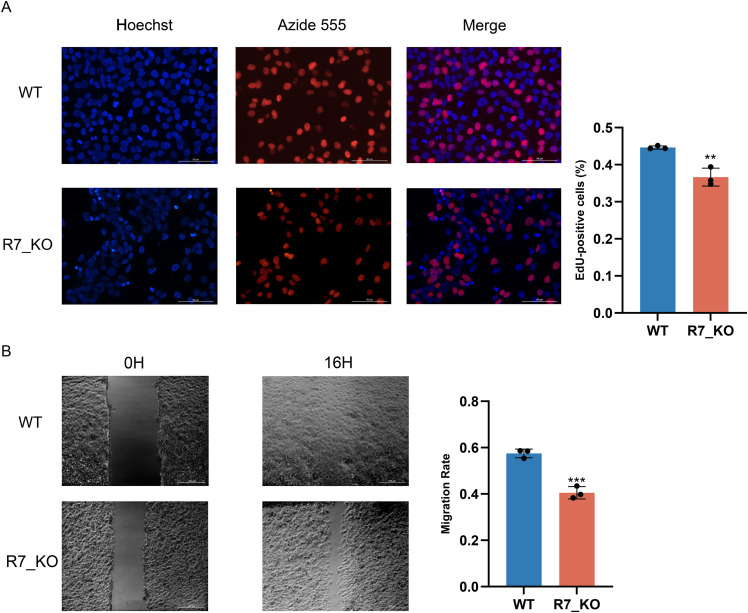
R7 deletion reduces EdU incorporation and cell migration in AC16 cells. **(A)** Representative EdU incorporation images of WT and R7_KO AC16 cells. Nuclei were stained with Hoechst, and EdU-positive cells were detected by azide 555 staining. Quantification of EdU-positive cells is shown on the right. Scale bars, 50 μm. **(B)** Representative wound-healing images of WT and R7_KO AC16 cells at 0 and 16 h after scratching. Quantification of wound closure/migration rate is shown on the right. Scale bars, 200 μm. Data are shown as mean ± SD. Statistical significance was determined by a two-tailed unpaired *t* test. **P < 0.01, ***P < 0.001.

To assess the cellular effects of R7 deletion and determine whether restoration of *JAG1* expression could alleviate these effects, we compared WT cells, R7_KO cells, and R7_KO cells transfected with different amounts of *JAG1* expression plasmid. The condition that restored *JAG1* mRNA to a level comparable with WT cells was designated *JAG1*-RE (5 ng/well), whereas the condition that resulted in *JAG1* expression above the WT level was designated *JAG1*-OE (50 ng/well) (Fig 2A). Cell-cycle analysis showed that R7_KO cells exhibited an increased proportion of cells in the G2/M phase compared with WT cells, indicating impaired cell-cycle progression (Fig 2B–D). Both *JAG1*-RE and *JAG1*-OE reduced G2/M accumulation in R7_KO cells, suggesting that restoration of *JAG1* expression can partially alleviate the cell-cycle defect associated with R7 deletion. We next assessed cell viability using the Cell Counting Kit-8 (CCK-8) assay. R7_KO cells showed reduced cell viability compared with WT cells over the time course examined (Fig 2E). *JAG1*-RE produced a modest improvement in cell viability, whereas *JAG1*-OE showed a stronger trend toward restoration and approached the WT level at later time points. Annexin V/PI staining further showed that R7_KO cells had an increased proportion of apoptotic cells compared with WT cells (Fig 2F and G). Both *JAG1*-RE and *JAG1*-OE reduced apoptosis in R7_KO cells, indicating that restoration of *JAG1* expression partially alleviates the pro-apoptotic phenotype associated with R7 deletion. In addition, 5-ethynyl-2′-deoxyuridine (EdU) incorporation assays showed reduced proliferative capacity in R7_KO cells compared with WT cells (Fig S1A), and wound-healing assays showed reduced migratory capacity after R7 deletion (Fig S1B). Together, these results show that R7 deletion impairs cell-cycle progression, reduces cell viability, and increases apoptosis in AC16 cells and that restoration of *JAG1* expression partially mitigates these cellular defects.

### R7 deletion alters local chromatin interactions surrounding the *JAG1* locus

**Figure 3.**
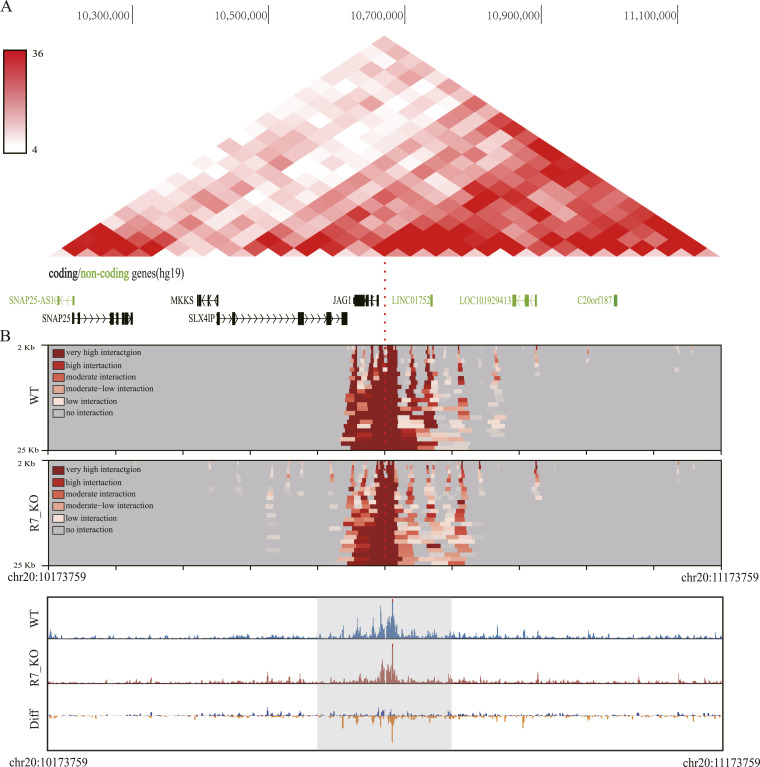
R7 deletion alters local chromatin contacts at the *JAG1* locus. **(A)** In situ Hi-C interaction heatmaps from human right ventricular tissue across a ±500 kb region centered on the *JAG1* locus, showing the local TAD containing JAG1. **(B)** 4C-seq profiles anchored at a viewpoint approximately 17 kb upstream of the *JAG1* TSS in WT and R7_KO AC16 cells. Tracks show viewpoint-specific contact signals in WT and R7_KO cells, together with the difference track (Diff), across the *JAG1* locus.

To determine whether R7 contributes to local chromatin topology near the *JAG1* locus, we compared chromatin interactions between WT and R7_KO AC16 cells. In situ high-throughput chromosome conformation capture (Hi-C) data from human right ventricular tissue showed that the *JAG1* locus resides within a topologically associating domain (TAD), with strong interaction signals extending across the upstream region of the *JAG1* transcription start site (Fig 3A). Circular chromosome conformation capture sequencing (4C-seq) interaction profiles generated from the r3Cseq-derived bedGraph data further showed that contact signals between the anchor point located approximately 17 kb upstream of the *JAG1* TSS and the surrounding 1-Mb region, including the *JAG1* promoter and upstream regulatory sequences, were reduced in R7_KO cells compared with WT cells (Fig 3B). These results indicate that R7 deletion alters local chromatin interactions and perturbs the 3D regulatory architecture surrounding the *JAG1* locus.

### R7 deletion reduces chromatin accessibility at CTCF-associated regions and is associated with cardiac-related transcriptional programs

**Figure 4.**
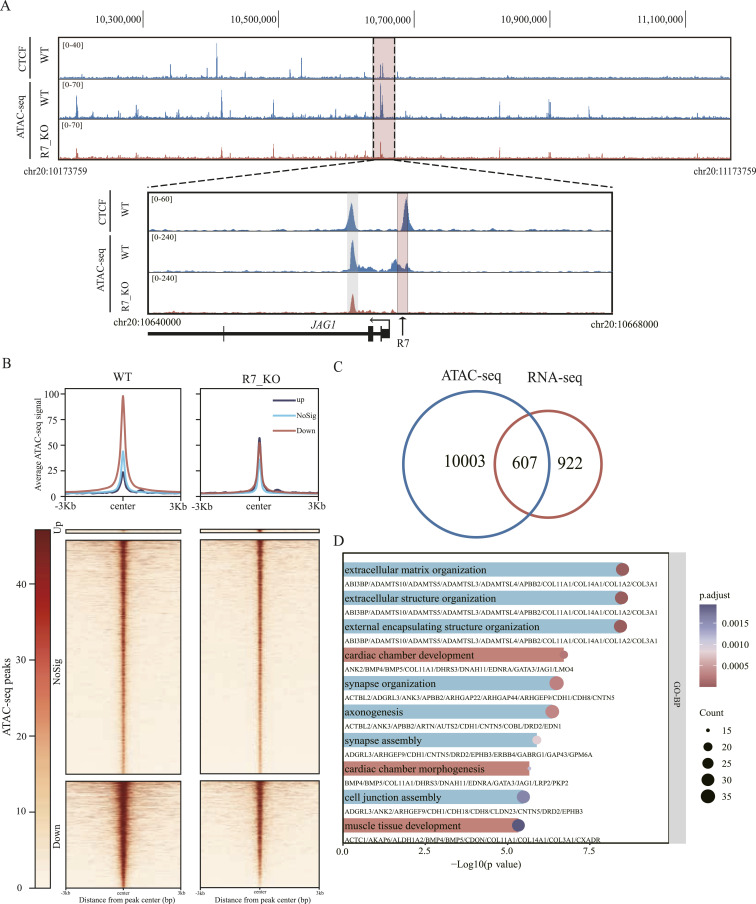
R7 deletion reduces local chromatin accessibility and is associated with heart-related biological processes. **(A)** CTCF ChIP-seq and ATAC-seq profiles across the *JAG1* locus in WT and R7_KO AC16 cells. In WT cells, overlapping CTCF-binding and ATAC-seq signals were mainly observed around R7 and within the second intron of *JAG1*. After R7 deletion, the broader ATAC-seq signal surrounding R7 was reduced, including the adjacent promoter-proximal region and the second intron. **(B)** Line plots and heatmaps showing ATAC-seq signal intensity within ±3 kb of peak centers in WT and R7_KO AC16 cells. Differentially accessible peaks were grouped as increased (Up), decreased (Down), or non-significant (NoSig). **(C)** Venn diagram showing the overlap between genes associated with differentially accessible ATAC-seq peaks and differentially expressed genes identified by RNA-seq in R7_KO versus WT cells. **(D)** Gene Ontology Biological Process (GO-BP) enrichment analysis of the overlapping genes shown in (C). The top 10 significantly enriched GO-BP terms ranked by adjusted *P*-value are shown, with cardiac-associated terms highlighted in red. Signal intensities are normalized as reads per kilobase per million mapped reads (RPKM).

**Figure S2.**
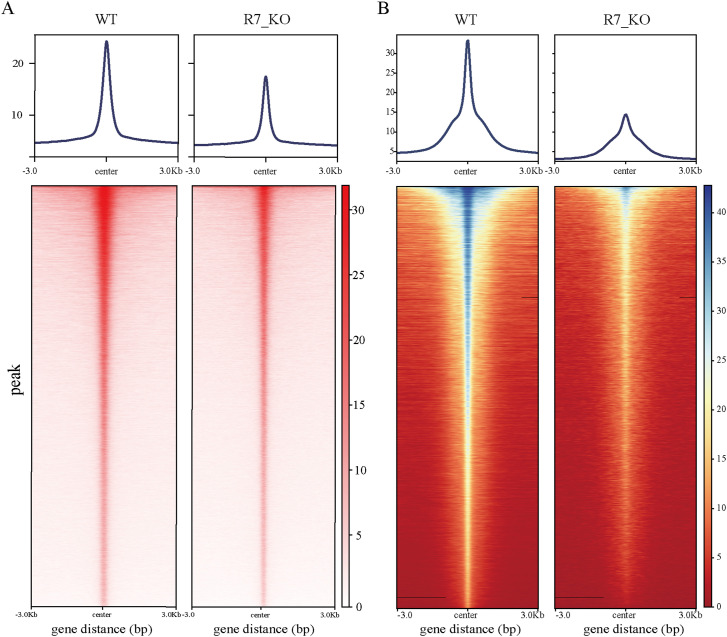
R7 deletion is associated with reduced genome-wide ATAC-seq and H3K27ac signal intensity. **(A)** Aggregate plot and heatmap of ATAC-seq signal showing a global reduction in chromatin accessibility in R7_KO cells compared with WT cells. **(B)** Aggregate plot and heatmap of H3K27ac ChIP-seq signal showing reduced H3K27ac signal intensity after R7 deletion.

To further assess the effect of R7 deletion on chromatin accessibility, we performed an assay for transposase-accessible chromatin using sequencing (ATAC-seq) analysis. Within the approximately 1-Mb window spanning the *JAG1* locus, overlapping ATAC-seq peaks and CTCF-binding signals in WT AC16 cells were largely confined to the region surrounding R7 and the second intron of *JAG1* (Fig 4A). After R7 deletion, the broader ATAC-seq signal surrounding the R7 region was markedly reduced, including the signal in the flanking promoter-proximal region adjacent to R7 and the signal within the second intron of *JAG1* (Fig 4A). Heatmap and profile analyses centered on differentially accessible regions showed an overall reduction in ATAC-seq signal intensity in R7_KO cells compared with WT cells (Fig 4B). In addition, aggregate peak-centered analysis showed reduced genome-wide ATAC-seq signal intensity in R7_KO cells (Fig S2A). To explore the biological relevance of these accessibility changes, we intersected genes associated with differentially accessible ATAC-seq peaks with differentially expressed genes identified by RNA-seq in R7_KO versus WT cells (Fig 4C). Gene Ontology Biological Process (GO-BP) enrichment analysis of the overlapping genes revealed significant enrichment of cardiac-related terms, including cardiac chamber development, cardiac chamber morphogenesis, and muscle tissue development (Fig 4D). Together, these results suggest that R7 deletion reduces chromatin accessibility at the *JAG1* locus and is associated with transcriptional changes in pathways relevant to cardiac development.

### R7 deletion alters transcription-associated chromatin features at the *JAG1* locus

**Figure 5.**
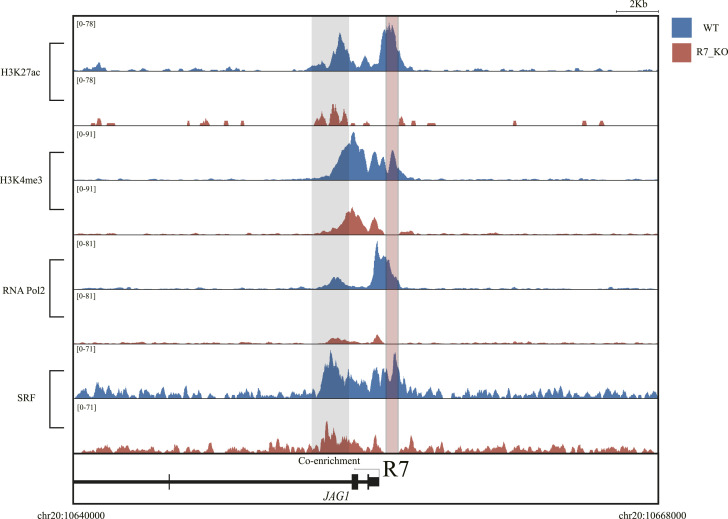
R7 deletion alters transcription-associated chromatin features at the *JAG1* locus. ChIP-seq signal tracks for H3K27ac, H3K4me3, RNA polymerase II (Pol II), and SRF in WT and R7_KO AC16 cells across the *JAG1* locus. In WT cells, these transcription-associated signals were enriched around the R7-associated promoter-proximal region and within the second intron of *JAG1*. After R7 deletion, H3K27ac, H3K4me3, Pol II occupancy, and SRF binding were reduced across this promoter–intron regulatory region. Signal intensities are normalized to RPKM.

**Figure S3.**
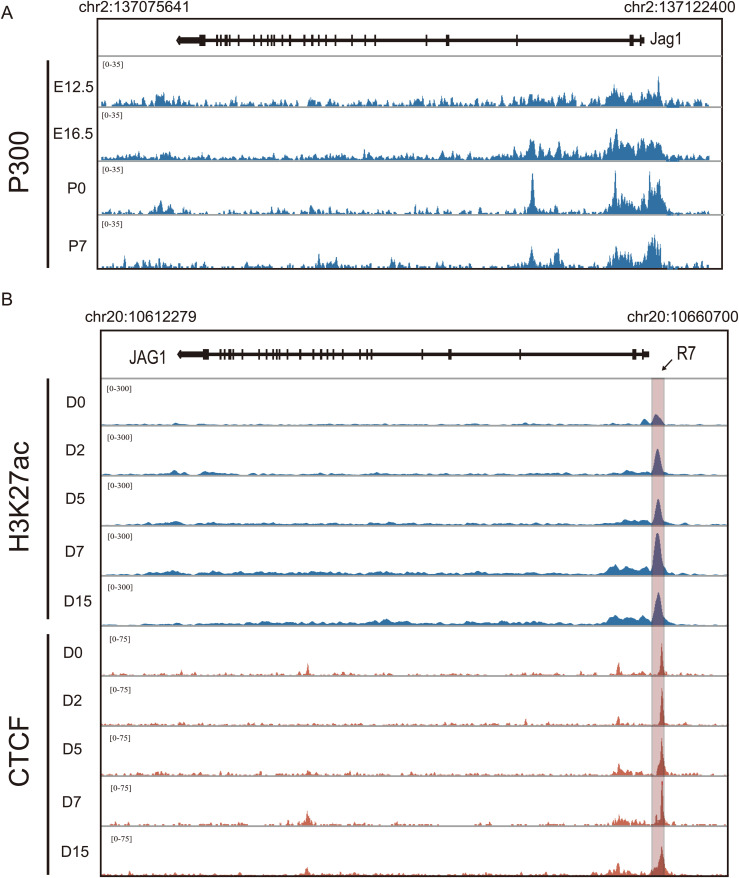
Public cardiac developmental ChIP-seq datasets show regulatory chromatin signals near the mouse Jag1 and human JAG1 loci. **(A)** Public p300 ChIP-seq profiles around the mouse Jag1 locus across the indicated cardiac developmental stages (GSE195901). E indicates embryonic day, and P indicates postnatal day. **(B)** Public H3K27ac and CTCF ChIP-seq profiles around the human JAG1 locus during iPSC-derived cardiomyocyte differentiation (GSE116862). D indicates differentiation day after initiation of cardiac differentiation. The shaded region indicates the R7-containing promoter-proximal region.

To further investigate the relationship between R7 and transcription-associated chromatin features at the *JAG1* locus, we performed ChIP-seq analysis of H3K27ac, H3K4me3, RNA polymerase II (Pol II), and SRF in WT and R7_KO AC16 cells. In WT cells, H3K27ac, a marker of active regulatory regions, was enriched around the promoter-proximal region containing R7 and within the second intron of *JAG1*. After R7 deletion, the H3K27ac signal was markedly reduced across the broader region surrounding R7 and within the second intron, suggesting that R7 deletion attenuates active chromatin features across the local promoter-proximal and intronic regulatory region (Fig 5). Consistently, aggregate peak-centered analysis also showed reduced genome-wide H3K27ac ChIP-seq signal intensity in R7_KO cells (Fig S2B). H3K4me3, typically associated with active promoters, was detected at the *JAG1* TSS and the second intron in WT cells. Upon R7 deletion, the H3K4me3 signal was reduced more prominently at the second intron, whereas the canonical promoter signal was less affected (Fig 5). Pol II occupancy at the *JAG1* promoter was also reduced in R7_KO cells, consistent with decreased *JAG1* transcription after R7 deletion (Fig 5). Given previous mouse cardiac ChIP-seq datasets mapping SRF occupancy together with other cardiac transcription factors at regulatory elements in mouse hearts (Akerberg et al, 2019), we examined SRF occupancy in AC16 cells. In WT cells, SRF occupancy was observed around the R7-containing promoter-proximal region and within the second intron of *JAG1*. After R7 deletion, SRF occupancy was reduced across this promoter-proximal and intronic regulatory region, suggesting that R7 may contribute to maintaining local SRF occupancy (Fig 5). Consistently, public cardiac developmental ChIP-seq datasets showed regulatory chromatin signals around the mouse *Jag1* and human *JAG1* loci. Enrichment of p300 was observed around the mouse *Jag1* locus across cardiac developmental stages (GSE195901) (Zhou et al, 2023; Fig S3A), whereas enrichment of H3K27ac and CTCF was observed at the R7-containing promoter-proximal region of the human *JAG1* locus during iPSC-derived cardiomyocyte differentiation (GSE116862) (Zhang et al, 2019; Fig S3B). Taken together, these data indicate that R7 deletion alters active chromatin marks, Pol II occupancy, and SRF binding at the *JAG1* promoter–intron region, thereby perturbing a transcription-permissive regulatory environment at the *JAG1* locus.

## Discussion

In this study, we used an integrative enhancer-associated screening strategy to identify heart-associated candidate regulatory regions surrounding the *JAG1* locus. Among these candidates, R7 showed the strongest reporter activity and, because of its close proximity to the *JAG1* transcription start site, was defined as a promoter-proximal regulatory element with enhancer-like activity. R7 deletion reduced *JAG1* expression and impaired AC16 cell phenotypes, which were partially alleviated by restoration of *JAG1* expression. Mechanistically, R7 deletion was associated with altered local chromatin contacts, reduced chromatin accessibility, and decreased transcription-associated chromatin features at the *JAG1* locus. Together, these findings suggest that R7 contributes to *JAG1* transcription by integrating promoter-proximal chromatin architecture with transcriptional regulatory activity.

Previous cardiac epigenomic studies have shown that enhancer-associated chromatin features provide valuable entry points for identifying heart-associated regulatory regions, including candidate cardiac enhancers and dynamic regulatory elements linked to cardiac gene expression programs (May et al, 2011; Wamstad et al, 2012). In this context, our R-panel-based screening prioritized R7 as the most active candidate region at the *JAG1* locus. However, R7 is located only 253 bp upstream of the *JAG1* transcription start site, placing it within a promoter-proximal genomic context. Current enhancer annotation frameworks emphasize that candidate regulatory elements should be interpreted by integrating reporter activity, endogenous genomic context, and target-gene linkage rather than by reporter activity alone (Gasperini et al, 2020). Consistently, although R7 retained reporter activity in the reverse orientation, supporting enhancer-like activity, it also drove reporter expression without the E1b minimal promoter, indicating promoter-like reporter activity. These findings are consistent with the broader view that enhancer and promoter functions are not strictly dichotomous and that promoter-proximal or promoter-associated regions can display enhancer-like regulatory properties (Vernimmen & Bickmore, 2015; Dao & Spicuglia, 2018). We, therefore, describe R7 as a promoter-proximal regulatory element with enhancer-like activity, rather than assigning it solely as an enhancer. Although this study focused on R7 because it showed the strongest reporter activity, R5 also showed significant reporter activity and may represent another candidate regulatory region for future perturbation-based evaluation. More broadly, the R-panel-defined candidate intervals may provide a useful framework for integrating reported patient noncoding variants near *JAG1*, thereby helping to prioritize regulatory regions with potential clinical relevance in CHD contexts.

The cellular phenotypes observed after R7 deletion are consistent with the established role of *JAG1*/Notch signaling in cardiac development. Myocardial *JAG1* regulates ventricular chamber development, and myocardium-specific *JAG1* knockout impairs compact myocardium proliferation during embryogenesis (D'Amato et al, 2016). In our study, R7 deletion reduced *JAG1* expression and was associated with impaired cell-cycle progression, reduced cell viability, and increased apoptosis in AC16 cells. Restoration of *JAG1* expression to near-WT levels partially alleviated the cell-cycle and apoptosis defects caused by R7 deletion. In the CCK-8 assay, *JAG1* restoration improved cell viability but did not fully restore it to the WT level, whereas *JAG1* overexpression produced a more evident recovery at later time points. Together, these findings support that reduced *JAG1* expression contributes to the cellular phenotypes caused by R7 deletion.

The chromatin-based analyses further suggest that R7 is functionally embedded within the local regulatory architecture of the *JAG1* locus. Rather than acting solely as a reporter-active sequence, R7 appears to contribute to a promoter-proximal chromatin environment in which local contacts, chromatin accessibility, and CTCF-associated regulatory features are coordinated. This interpretation is supported by the reduced 4C contact signals after R7 deletion and by the attenuation of the broader ATAC-seq signal surrounding R7, including the adjacent promoter-proximal region and the second intron of *JAG1*. Such findings are consistent with the concept that promoter-associated chromatin contacts can participate in transcriptional regulation and may be dynamically remodeled across cellular states (Siersbaek et al, 2017). Given the established role of CTCF in organizing local chromatin structure and regulatory insulation (Del Moral-Morales et al, 2023), the overlap between CTCF binding and accessible chromatin around R7 and the second intron suggests that R7 may help maintain a local architecture permissive for *JAG1* transcription.

The coordinated changes in active chromatin marks, Pol II occupancy, and SRF binding suggest that the R7-associated promoter–intron region functions as a local regulatory module at the *JAG1* locus. This interpretation is supported by previous work showing that active promoters are frequently embedded in promoter-centered chromatin interaction networks involving intragenic and nearby regulatory regions, thereby providing a topological framework for transcriptional regulation (Li et al, 2012). In addition, promoter-associated elements with enhancer-like activity have been shown to regulate gene expression in their endogenous genomic context, with perturbation of these elements affecting target-gene expression, local chromatin contacts, and active chromatin features (Dao et al, 2017). In our study, R7 deletion was accompanied by reduced H3K27ac across the R7-associated promoter-proximal region and the second intron, decreased H3K4me3 and Pol II occupancy at the *JAG1* promoter, and reduced SRF occupancy across the same promoter–intron regulatory region. Because SRF was selected based on published murine cardiac TF occupancy maps (Akerberg et al, 2019), these data suggest that R7 may help maintain local cardiac TF occupancy within this regulatory module, although whether SRF directly mediates the regulatory effect of R7 requires further perturbation-based validation. These findings support a model in which R7 integrates local transcription factor binding, active chromatin features, and *JAG1* transcriptional output. Independent public cardiac developmental ChIP-seq datasets further support this regulatory context, showing p300 enrichment near the mouse *Jag1* locus and enrichment of H3K27ac and CTCF at the human R7-containing promoter-proximal region during iPSC-derived cardiomyocyte differentiation. Nevertheless, these epigenomic data do not replace direct functional validation in developmental or in vivo models.

Several limitations should be considered when interpreting these findings. First, because R7 is located in proximity to the *JAG1* TSS and displays both enhancer-like and promoter-like reporter properties, its precise endogenous regulatory identity remains to be further defined. Future studies will be needed to distinguish the relative contributions of enhancer-like activity, promoter-proximal regulation, and possible promoter-like function at the endogenous *JAG1* locus. Second, although public cardiac developmental epigenomic datasets support a regulatory context for the R7-containing region, the physiological function of R7 remains to be directly validated in developmental and in vivo models. Third, because the functional experiments were mainly performed in a single R7-deletion AC16 cell model, potential clone-specific or CRISPR-related effects cannot be fully excluded. R7 activity and function should, therefore, be further tested in independent genome-edited clones and additional cardiac and non-cardiac cellular contexts, including primary cardiomyocytes and iPSC-derived cardiomyocytes. Finally, the second intron of *JAG1* and SRF occupancy emerged as associated features of the R7-linked regulatory region, but their causal contributions remain to be tested.

Together, our study identifies R7 as a cardiac-associated promoter-proximal regulatory element that contributes to *JAG1* transcription through local chromatin architecture and transcription-associated regulatory features. These findings provide a defined regulatory interval for investigating noncoding regulation at the *JAG1* locus and may help prioritize candidate noncoding variants near *JAG1* in cardiac disease contexts.

## Materials and Methods

### Identification and clustering of candidate enhancer regions

To systematically identify candidate heart-associated enhancer regions near the *JAG1* locus, we followed the previously described R-panel pipeline for integrative heart-specific enhancer prediction (Wang et al, 2022). The R-panel was constructed from 148 public ChIP-seq peak datasets covering three regulatory histone marks, H3K4me1, H3K4me3, and H3K27ac, across heart, liver, kidney, and brain tissues from human and mouse samples. These datasets were obtained from Gene Expression Omnibus (GEO) (Edgar et al, 2002) and Encyclopedia of DNA Elements (ENCODE) (https://www.encodeproject.org/) through Cistrome DB (Mei et al, 2017). Datasets with unhealthy or disease-related sample annotations were excluded. Only datasets passing the quality-control criteria used in the original R-panel pipeline were retained; specifically, each dataset met at least three Cistrome DB quality-control metrics, including sequencing quality, uniquely mapped reads, PCR bottleneck coefficient, peak number/enrichment, fraction of reads in peaks, genomic distribution of peaks, and overlap with DNase I hypersensitive sites. Peak coordinates originally mapped to hg38 or mm10 were converted to the human hg19 genome assembly using LiftOver. For mouse-to-human conversion, a minimum remapping ratio of 0.5 was used, and peaks with a length-change ratio greater than 1 after LiftOver were excluded. The retained datasets were then grouped by species and histone mark into six categories: human H3K4me1, human H3K4me3, human H3K27ac, mouse H3K4me1, mouse H3K4me3, and mouse H3K27ac. Within each group, heart-associated enrichment scores were calculated by comparing peak occurrence at each genomic position in heart samples with that in non-cardiac control tissues, followed by normalization to an importance-score scale ranging from 0 to 1,000. Candidate peaks located within a ±100 kb window surrounding the *JAG1* locus with scores ≥749 were retained for further analysis. Adjacent retained peaks with an inter-peak distance <1,000 bp were merged into candidate regions, generating the R1–R10 regions used for luciferase-based functional screening.

### Culture, vector construction, and luciferase assay

AC16 human cardiomyocyte cells were cultured in Dulbecco’s Modified Eagle’s Medium (Gibco) supplemented with 10% fetal bovine serum (Gibco) and 1% penicillin/streptomycin (Gibco) at 37°C in a humidified incubator containing 5% CO_2_. Candidate enhancer fragments were amplified by PCR from HEK293 genomic DNA using 2× Phanta Flash Master Mix (Vazyme). Primers are listed in Table S1 and were designed to flank the predicted candidate regions, with slight extension beyond the target boundaries when needed to accommodate potential high GC content. The amplified candidate fragments were cloned upstream of the E1b minimal promoter in the pGL3-E1b luciferase vector through the KpnI and XhoI sites and assembled using 2× ClonExpress Mix (Vazyme). To further evaluate the regulatory properties of R7, two additional reporter constructs, R7_Reverse and R7_ΔE1b, were generated by Tsingke Biotechnology Co., Ltd. R7_Reverse contains the same R7 fragment inserted upstream of the E1b minimal promoter in the opposite genomic orientation relative to the forward R7 construct, whereas R7_ΔE1b contains R7 cloned directly upstream of the luciferase coding sequence after removal of the E1b minimal promoter. For luciferase reporter assays, AC16 cells were seeded in 96-well plates and cultured to ∼70–80% confluency. Transfections were performed using jetPRIME DNA transfection reagent (Polyplus) according to the manufacturer’s protocol. Each well was transfected with 100 ng of reporter vector and 10 ng of pRL-TK Renilla luciferase control vector (Promega). Luciferase activities were measured 48 h after transfection using the Dual-Luciferase Reporter Assay Kit (Vazyme) on a Varioskan LUX microplate reader (Thermo Fisher Scientific), and Firefly luciferase activity was normalized to Renilla luciferase activity. All assays were performed in biological triplicate.

Table S1. Primer sequences used for PCR amplification of candidate enhancer-associated fragments.

### Generation of R7-deletion AC16 cell lines

Single-guide RNAs (sgRNAs) targeting the R7 region were designed using the Red Cotton CRISPR design tool (https://en.rc-crispr.com/), based on specificity and efficiency scores. Because R7 is located close to the *JAG1* TSS, sgRNAs were selected from suitable target sites around the R7-containing region according to available PAM sequences and guide-design scores. The proximal sgRNA cut site was located approximately 228 bp upstream of the *JAG1* TSS. The selected oligonucleotide pairs were annealed and cloned into the YKO-RP006 vector (Ubigene Biosciences). The sgRNA target sequences for R7 are provided in Table S2. Recombinant YKO-RP006-R7 plasmids were transfected into AC16 cells using Lipofectamine 3000 (Thermo Fisher Scientific). At 24–48 h post-transfection, puromycin was added for selection. Surviving cells were diluted and seeded into 96-well plates using a limiting dilution approach. After 2–4 wk, single-cell clones were isolated and screened. R7 knockout clones (R7_KO) were validated by PCR and Sanger sequencing.

Table S2. Oligonucleotide sequences used for RT-qPCR, CRISPR/Cas9-mediated R7 deletion, and 4C-seq.

### Expression validation

Total RNA was extracted from AC16 cells using TRIzol Reagent (Invitrogen) according to the manufacturer’s protocol. Reverse transcription was performed using the PrimeScript RT reagent Kit (TaKaRa). Quantitative real-time PCR was performed in triplicate using TB Green Premix Ex Taq II (TaKaRa) on an ABI 7500 Real-Time PCR System (Applied Biosystems). GAPDH was used as the internal reference, and relative mRNA expression was calculated using the 2^−ΔΔCt method. Primer sequences used for RT-qPCR are provided in Table S2. For Western blot analysis, total protein was extracted from AC16 cells using RIPA buffer (Thermo Fisher Scientific) supplemented with protease inhibitors. Protein concentrations were measured using a BCA assay (Beyotime). Equal amounts of protein were separated on 10% precast SDS–PAGE gels (Epizyme) and transferred to PVDF membranes. Membranes were blocked in 5% non-fat milk and incubated overnight at 4°C with primary antibodies against JAG1 (Proteintech) and GAPDH (Proteintech), followed by HRP-conjugated secondary antibodies. Signals were visualized using enhanced chemiluminescence and captured with a ChemiDoc imaging system (Bio-Rad). Band intensities were quantified using ImageJ, and relative JAG1 protein expression was normalized to GAPDH.

### *JAG1* restoration and overexpression in R7_KO cells

To assess whether reduced *JAG1* expression contributes to the cellular phenotypes caused by R7 deletion, *JAG1* expression was restored or overexpressed in R7_KO AC16 cells by transient plasmid transfection. The human *JAG1* overexpression plasmid, human *JAG1* (NM_000214) pcDNA3.1-3×Flag-C, was purchased from Ubigene Biosciences. R7_KO cells were seeded in 24-well plates and transfected using jetPRIME DNA transfection reagent according to the manufacturer’s protocol. For the *JAG1* restoration group, designated JAG1-RE, cells were transfected with 5 ng of *JAG1* expression plasmid per well in 24-well plates, which restored *JAG1* expression to a level comparable with WT cells. For the JAG1 overexpression group, designated JAG1-OE, cells were transfected with 50 ng of *JAG1* expression plasmid per well in 24-well plates to achieve higher *JAG1* expression. Cells were collected 48 h after transfection for RT-qPCR, cell-cycle analysis, CCK-8 assay, and apoptosis analysis.

### RNA-seq

Total RNA was extracted from cells using TRIzol reagent. Briefly, 1 ml of TRIzol was added to the cell pellet, followed by 200 μl of chloroform. After vigorous shaking and centrifugation at 4°C, the aqueous phase was transferred to a new tube. RNA was precipitated with 500 μl isopropanol, pelleted by centrifugation, washed with 75% ethanol, and dissolved in DEPC-treated water. For library preparation, 4 μg of total RNA was processed using the Vazyme RNA Library Prep Kit (NRM605) according to the manufacturer’s protocol. Polyadenylated mRNA was enriched using oligo-dT magnetic beads and fragmented at an elevated temperature. First- and second-strand cDNA synthesis was carried out to generate double-stranded DNA, followed by adaptor ligation and PCR amplification. Libraries were sequenced on the MGI platform. Raw reads were filtered for quality using fastp (v0.23.4) (Chen et al, 2018). Clean reads were aligned to the human hg19 reference genome using the STAR aligner (v2.7.11b) (Dobin et al, 2013) with default parameters. Gene-level quantification was performed with featureCounts (v2.0.6) (Liao et al, 2014), and differential expression analysis was carried out using DESeq2 (v1.44.0) (Love et al, 2014), applying a cutoff of |log2 fold change| >1 and *P*-value <0.05. The clusterProfiler R package was used to perform GO enrichment, with the Biological Process (BP) ontology selected to identify functional categories enriched among differentially expressed genes (DEGs).

### Cell-cycle assay

For rescue and overexpression experiments, WT, R7_KO, JAG1-RE, and JAG1-OE cells were collected 48 h after transfection as described above. AC16 cells were digested with trypsin, collected by centrifugation, and washed once with PBS. Cells were fixed in 70% cold ethanol at 4°C overnight. After fixation, the cells were washed with PBS and stained according to the instructions of the Cell Cycle and Apoptosis Analysis Kit (Beyotime). DNA content was measured by flow cytometry, and the distribution of cell-cycle phases (G1, S, and G2/M) was analyzed using FlowJo software.

### CCK-8 assay

Cell viability was assessed using the Cell Counting Kit-8 (CCK-8; Dojindo Laboratories) according to the manufacturer’s instructions. AC16 cells were seeded into 96-well plates and transfected with the indicated *JAG1* expression plasmids after attachment. At 24 h after transfection, the first CCK-8 measurement was performed and defined as 0 h. Cells were then cultured for up to 4 d, and CCK-8 measurements were repeated at the indicated time points. For each measurement, CCK-8 reagent was added to each well, and cells were incubated at 37°C for 3 h. Absorbance was measured at 450 nm using a microplate reader, and relative cell viability was calculated based on the absorbance values.

### Apoptosis assay

For apoptosis analysis, WT, R7_KO, JAG1-RE, and JAG1-OE cells were collected 48 h after transfection as described above. AC16 cells from the indicated groups were digested with trypsin, collected by centrifugation, and washed twice with cold PBS. Cells were then resuspended in binding buffer and stained with Annexin V-FITC and PI using the Annexin V-FITC/PI Apoptosis Detection Kit (Biosharp) according to the manufacturer’s instructions. Staining was performed in the dark for 15 min at room temperature, and samples were analyzed by flow cytometry within 1 h. Early and late apoptotic populations were quantified using FlowJo software based on Annexin V and PI signal gating.

### EdU incorporation assay

AC16 cells were cultured to an appropriate density and incubated with EdU working solution for 2 h. Cells were then fixed in 4% paraformaldehyde and permeabilized with 0.3% Triton X-100 in PBS. Subsequent staining steps were carried out according to the protocol of the BeyoClick EdU-555 Cell Proliferation Kit (Beyotime), with Hoechst used for nuclear counterstaining. Fluorescence images were acquired using fluorescence microscopy, and representative images are shown with 50 μm scale bars. EdU-positive cells were quantified as the percentage of EdU-positive cells among total Hoechst-stained cells.

### Cell migration assay

AC16 cells were seeded into 6-well plates and cultured until reaching full confluency. A sterile pipette tip was used to generate a scratch across the cell monolayer. After washing twice with PBS to remove detached cells, serum-free medium was added, and cells were incubated for 16 h. Phase-contrast images of the wound area were acquired at 0 and 16 h using phase-contrast microscopy with a 10× objective lens. Representative images are shown with 200 μm scale bars. Wound closure was quantified using ImageJ by measuring the wound area at 0 and 16 h, and the migration rate was calculated as the percentage of wound closure relative to the initial wound area.

### Hi-C data analysis

We analyzed publicly available Hi-C data from human right ventricular tissue (GSE58752), originally generated and published in a prior epigenomic study (Leung et al, 2015). Raw contact matrices aligned to the hg19 reference genome were accessed via the 3D Genome Browser (https://3dgenome.fsm.northwestern.edu), and interaction profiles surrounding the *JAG1* locus were visualized using the browser’s integrated tools.

### 4C-seq

4C-seq was performed using two biological replicates for each genotype. The 4C-seq library was constructed according to a previously published protocol (Jia et al, 2020). Briefly, 500 million cells were first crosslinked with formaldehyde and then digested with the restriction enzyme DpnII. The resulting chromatin fragments were re-ligated using T4 DNA ligase to capture spatially proximal regions. The ligated DNA was subsequently purified, sheared by sonication, and linearly amplified with incorporation of biotin-labeled nucleotides. Biotinylated DNA was enriched using streptavidin-coated magnetic beads. After adaptor ligation and PCR amplification, the library was sequenced using the Illumina platform. The sequences of primers used for 4C-seq are listed in Table S2. Raw sequencing reads were processed with Cutadapt (v5.0) (Kechin et al, 2017) to remove adapters and barcodes. High-quality reads were aligned to the hg19 human reference genome using Bowtie2 (v2.2.5) (Langmead & Salzberg, 2012) with default parameters. Interaction analysis was performed using the r3Cseq (v1.50.0) (Thongjuea et al, 2013) pipeline, focusing on statistically significant contacts anchored to the predefined viewpoint.

### ATAC-seq

ATAC-seq was performed using two biological replicates for each group. ATAC-seq libraries were constructed using the Hyperactive ATAC-Seq Library Preparation Kit for Illumina (TD711; Vazyme) with 50,000 cells per sample. Cells were lysed in lysis buffer to isolate nuclei, which were then incubated with Tn5 transposase at 37°C for 30 min to fragment the genomic DNA. Transposed DNA was purified using ATAC DNA extraction beads. The resulting DNA fragments were PCR-amplified to generate sequencing libraries, followed by two rounds of size selection using the same magnetic bead system. Sequencing reads were processed using Fastp (v0.23.4) to remove low-quality bases and adaptor sequences. Clean reads were aligned to the human hg19 reference genome using Bowtie2 (v2.2.5). PCR duplicates were removed with Picard, and accessible chromatin peaks were called using MACS2 (v2.2.7.1) (Feng et al, 2012) with default parameters. Differential peak analysis between WT and R7_KO groups was performed using DESeq2 (v1.44.0). Peaks were categorized as increased or decreased based on the sign of the DESeq2 log2 fold change, with false discovery rate (FDR) <0.05 used as the significance cutoff; peaks with FDR ≥0.05 were classified as non-significant. For integrated functional analysis, genes associated with differentially accessible ATAC-seq peaks were intersected with differentially expressed genes identified by RNA-seq. The overlapping genes were subjected to Gene Ontology Biological Process enrichment analysis using clusterProfiler, and the top 10 enriched terms ranked by adjusted *P*-value were displayed.

### Candidate TF identification

The cardiac transcription factor SRF was selected as a candidate regulator based on previously published bioChIP-seq datasets profiling cardiac-enriched TFs in fetal and adult mouse hearts (GSE124008) (Akerberg et al, 2019). The human R7 region and the *JAG1* second-intron region were lifted over to the mouse genome, and TF occupancy at the corresponding murine loci was examined. SRF was selected as a candidate factor based on its occupancy in the cardiac TF bioChIP-seq dataset.

### ChIP-seq

ChIP-seq was performed using two biological replicates for each condition and target. For SRF ChIP-seq, AC16 cells were transfected with a plasmid encoding 3×FLAG-tagged SRF, and samples were collected 48 h post-transfection. Immunoprecipitation was performed using anti-FLAG antibody (Proteintech). For all other ChIP-seq experiments, including those targeting H3K27ac, H3K4me3, RNA polymerase II, and CTCF, endogenous proteins were enriched using target-specific antibodies (Active Motif). Cells were fixed in 1% formaldehyde for 10 min, and the reaction was terminated with 0.125 M glycine. After washing with cold PBS containing protease inhibitors, cells were scraped, collected, and lysed in buffer I (50 mM HEPES-NaOH pH 7.5, 300 mM NaCl, 1 mM EDTA, 0.1% sodium deoxycholate, 1% Triton X-100, 0.025–0.2% SDS, protease inhibitors). Chromatin was fragmented by sonication and centrifuged to collect soluble chromatin. Chromatin was incubated with the antibody overnight at 4°C. Protein A/G magnetic beads (Thermo Fisher Scientific) were pre-washed with buffer I and incubated with the antibody–chromatin complex. Beads were sequentially washed with buffer I, LiCl buffer (10 mM Tris–HCl pH 8.0, 250 mM LiCl, 1 mM EDTA, 0.5% NP-40, 0.5% sodium deoxycholate), and TE buffer (50 mM Tris–HCl pH 8.0, 10 mM EDTA, 50 mM NaCl). Chromatin was eluted with decrosslink buffer (50 mM Tris–HCl pH 8.0, 10 mM EDTA, 1% SDS), followed by enzymatic digestion in decrosslink dilution buffer (50 mM Tris–HCl pH 8.0, 10 mM EDTA, 50 mM NaCl) with RNase A and Proteinase K (TransGen Biotech). DNA was purified using the QIAquick PCR Purification Kit (Qiagen) and subjected to next-generation sequencing. Raw reads were trimmed and quality-filtered using Trim Galore and aligned to the hg19 human genome using Bowtie2. Normalized signal tracks were generated with deepTools (v3.5.6) (Ramirez et al, 2016), using RPKM normalization, to generate normalized bigWig signal tracks for WashU browser visualization.

### Statistical analysis

Data are presented as mean ± SD. For two-group comparisons, statistical significance was assessed using a two-tailed unpaired *t* test. For multiple-group luciferase comparisons, one-way ANOVA followed by Dunnett’s multiple-comparisons test was used where indicated. *P* < 0.05 was considered statistically significant. Statistical analyses were performed using GraphPad Prism.

## Supplementary Material

Reviewer comments

## Data Availability

The raw sequencing data generated in this study, including RNA-seq, ATAC-seq, ChIP-seq, and 4C-seq datasets, have been deposited in the European Nucleotide Archive (ENA) at EMBL-EBI under accession number PRJEB102413. Public Hi-C data from human right ventricular tissue were obtained from the NCBI Gene Expression Omnibus under accession number GSE58752 and visualized through the 3D genome browser. Public murine cardiac transcription factor bioChIP-seq data used for candidate TF selection were obtained from GSE124008. Public cardiac developmental ChIP-seq datasets used for supplementary epigenomic analyses were obtained from GSE195901 and GSE116862. All other data supporting the findings of this study are available from the corresponding authors upon reasonable request.
