# Supplementary material for: A cardiac-related, promoter-proximal, regulatory element shapes chromatin and JAG1 transcription
Source: Life Sci Alliance. 2026 Aug 3;9(10):e202503597. doi: 10.26508/lsa.202503597 (PMC13433824; doi:10.26508/lsa.202503597)
Supplement: Supplementary file 1 [file LSA-2025-03597_review_history.pdf]

A cardiac-related, promoter-proximal, regulatory element shapes chromatin and JAG1 transcription

Quannan Zhuang, Feng WANG, Huinan Zhang, Qi Zhang, Yuan Gao, Zhiyu Feng, Han Gao, Siyu Sun, Siyi Lin, Shuolin Li, Quming Zhao, Xianghui Huang, Qiang Li, Wei Sheng, and Guoying Huang
DOI: https://doi.org/10.26508/lsa.202503597

Corresponding author(s): Guoying Huang, Children's Hospital of Fudan University; Wei Sheng, Children's Hospital of Fudan University and Qiang Li, Children's Hospital of Fudan University

Review Timeline:

Submission Date:	2025-12-15
Editorial Decision:	2026-03-03
Revision Received:	2026-05-13
Editorial Decision:	2026-06-22
Revision Received:	2026-07-02
Accepted:	2026-07-03

Scientific Editor: Sarita Hebbar

Transaction Report:

(Note: With the exception of the correction of typographical or spelling errors that could be a source of ambiguity, letters and reports are not edited. The original formatting of letters and referee reports may not be reflected in this compilation.)

March 3, 2026

Re: Life Science Alliance manuscript #LSA-2025-03597-T

Prof. Guoying Huang
Fudan University
China

Dear Dr. Huang,

Thank you for submitting your manuscript entitled "A Cardiac-Associated Enhancer Near the JAG1 Locus Regulates Chromatin Architecture and Transcriptional Activity" to Life Science Alliance. We apologise for the delay in communicating our decision due to reviewer availability.

Your manuscript was assessed by two reviewers whose comments are appended below. As you will note, the reviewers have acknowledged your efforts in the identification of a regulatory element of the Jag1 locus and the outcome of its deletion in A16 cells. That said we agree that significant concerns raised by the reviewers preclude publication of this work for now. Based on their overall evaluation, we invite you to submit a significantly revised manuscript addressing the reviewers' concerns.

We bring your attention to the concerns of both reviewers on the conclusion that R7 is an enhancer (Reviewer 1, point 2, and Reviewer 3, see points 1,3,5). We concur with these reviewers that a revised manuscript must include new data for functional evidence (Reviewer 1). Further you must provide a clear explanation for qualification for R7 as an enhancer whilst considering the point of Reviewer 3 on R7 acting as an alternative promoter (under point 5). Further you must comment on its unusual close proximity to the TSS as explained by Reviewer 3 (under additional issue) with appropriate mention in the title as well. Related to this, we highlight you to the point of Reviewer 1, points 3 and 4 on the lack any physiological evidence of R7 in cardiac development or physiology. We agree that you must discuss these limitations in your revised manuscript.

When submitting the revision, please include a letter addressing the reviewers' comments point by point. While a rebuttal must respond to all points in some form, additional experiments to resolve these points, other than indicated above, are not required.

I would be happy to discuss the revision in more detail via email or phone/videoconferencing. Please let me know which option you prefer, if any.

To upload the revised version of your manuscript, please log in to your account: https://lsa.msubmit.net/cgi-bin/main.plex

You will be guided to complete the submission of your revised manuscript and to fill in all necessary information. Please get in touch in case you do not know or remember your login name.

The typical timeframe for revisions is three months. Please note that papers are generally considered through only one revision cycle, so strong support from the referees on the revised version is needed for acceptance.

While you are revising your manuscript, please also attend to the below editorial points to help expedite the publication of your manuscript. Please direct any editorial questions to the journal office.

Thank you for this interesting contribution to Life Science Alliance. We hope that the comments below will prove constructive as your work progresses, and we are looking forward to receiving your revised manuscript.

Sincerely,

Sarita Hebbar, PhD
Scientific Editor
Life Science Alliance
http://www.lsajournal.org

A. THESE ITEMS ARE REQUIRED FOR REVISIONS

-- A letter addressing the reviewers' comments point by point.

-- An editable version of the final text (.DOC or .DOCX) is needed for copyediting (no PDFs).

-- High-resolution figure, supplementary figure and video files uploaded as individual files: See our detailed guidelines for preparing your production-ready images, https://www.life-science-alliance.org/authors

-- Summary blurb (enter in submission system): A short text summarizing in a single sentence the study (max. 200 characters including spaces). This text is used in conjunction with the titles of papers, hence should be informative and complementary to the title and running title. It should describe the context and significance of the findings for a general readership; it should be written in the present tense and refer to the work in the third person. Author names should not be mentioned.

-- By submitting a revision, you attest that you are aware of our payment policies found here: https://www.life-science-alliance.org/copyright-license-fee

B. MANUSCRIPT ORGANIZATION AND FORMATTING:

Full guidelines are available on our Instructions for Authors page, https://www.life-science-alliance.org/authors

We encourage our authors to provide original source data, particularly uncropped/-processed electrophoretic blots and spreadsheets for the main figures of the manuscript. If you would like to add source data, we would welcome one PDF/Excel-file per figure for this information. These files will be linked as supplementary "Source Data" files.

IMPORTANT: It is Life Science Alliance policy that if requested, original data images must be made available. Failure to provide original images upon request will result in unavoidable delays in publication. Please ensure that you have access to all original microscopy and blot data images before submitting your revision.

Reviewer #1 (Comments to the Authors (Required)):

In this report, cardiac-specific enhancers for Jag1 gene were investigated first through integrated analysis of the public genomic/epigenomic datasets, followed by reporter assay (positive) and deletion in AC16 cells. The transcriptional and cellular impact of enhancer deletion were evaluated in AC16 wildtype and deletion mutant cells. Additional impact on epigenetic features and chromatin architecture was evaluated by ChiP seq, ATAC-seq and HiC. Finally, a candidate transcription factor SRF was proposed. The authors concluded from these results that an SRF occupying enhancer for JAG1 was detected, implicating potential mechanism of SRF mediated regulation for JAG1 expression.

JAG1 is an important regulator in development with a major role in fetal cardiovascular differentiation and growth. Uncover the regulatory mechanism for JAG1 expression through enhancer identification would be valuable. However, there are major limitations in this study.

1). The R-pipeline used to identify the initial cardiac-specific enhancers for JAG1 should be carefully described in detail, with all the parameters and filtering criteria leading to the selection of the final 19 candidates.

2). Authors used AC16 cells to demonstrate tissue-specificity but failed to provide a non-myocyte cell line as a key control. The enhancer AC16 cells were extensively characterized, but the functional impact should be validated by re-storing JAG1 expression through an ectopic expression vector. This is a very important control to support that all the observed phenotypic changes are indeed caused by the loss of JAG1 expression.

3). Without any in vivo demonstration of the enhancer activity or the epigenetic/chromatin features of the enhancer locus in heart, it is not clear about the physiological validity of the enhancer activity in the context of cardiac development or physiology. AC16 is not a fully differentiated myocyte model. This is another major limitation of this study.

4). The authors presented only correlative and descriptive data on the SRF as a candidate trans-acting TF for JAG1 activity. It's contribution to local enhancer activity, chromatin and epigenetic reprogramming should be direct tested in Wildtype vs. KO cells using targeted KD or Over=expression approaches.

5). The authors presented only one AC16 enhancer KO cell line. Considering the potential off-target effects from Crispr based genome editing, it is important to show at least an alternative cell line for functional validation.

6). All bar-graph results should be presented with all data points in order to illustrate reproducibility of the data. Similar concerns about the epigenetic, chromatin profiling. It is unclear how many samples were analyzed and whether the differences illustrated were reproducible.

Reviewer #3 (Comments to the Authors (Required)):

1. Summary of the paper:
Non-coding patient variants mapping to, for example, enhancers, are likely to contribute to a significant amount of (congenital) disease cases, but causal patient variants are harder to identify due to a knowledge gap in the understanding of the cis-regulatory mechanisms regulating gene expression. The authors identify a relevant and interesting challenge in the field: How to best systematically screen for and prioritize enhancer candidates, and how to then functionally test them in order to identify the key regulatory elements that govern tissue-specific trancriptional regulation. After prioritizing enhancer candidates, they use a reporter assay for functional evaluation. From the 2 identified active regions, they chose to delete the strongest element in a human cell line model of cardiomyocytes and characterize the chromatin state, histone modifications, and 3D chromatin structure, identifying changes at the JAG1 locus and on a genone-wide level. They also observe cellular phenotypes that are in line with impaired centricular development in a murine Jag1 knockout model. The paper presents a comprehensive pipeline that spans enhancer prediction, epi-/genomic characterisation and functional testing, spanning the key steps to characterise regulatory elements and to prioritize genomic regions relevant to diagnose and potentially treat (congenital heart) disease.

2. Main points of the paper:

Point 1: Enhancer prediction pipeline and functional validation/prioritization using a dual-luciferase reporter assay.
The authors used their "previously established" R-panel to identify enhancers based on an integrated analysis pipeline of 148 ChiP-seq datasets from several tissues, including heart. Ten enhancers/enhancer clusters were identified for testing in a reporter assay and 2 sequences showed significantly stronger luciferase signal, indicating their ability to enhance target gene transcription. This is a solid strategy to efficiently screen a large genomic locus (+/- 100kb window). They then chose to continue with the detailed characterisation of "R7", which is located 253bp upstream of the JAG1 TSS.
The CRISPR/Cas9-deletion of R7 seems to have been intriduced by cutting even closer to the TSS than 253bps.

Major criticism: Typically, enhancers act at a distance of few kilobases to tens of kilobases (Gasperini, M., Tome, J.M. & Shendure, J. Towards a comprehensive catalogue of validated and target-linked human enhancers. Nat Rev Genet 21, 292-310 (2020). https://doi.org/10.1038/s41576-019-0209-0).
This review, for example, gives a cutoff of a distance of over 1000bp from the promoter for enhancers. See: "Genes transcribed by RNA polymerase (Pol)II usually have two distinct families of cis-acting elements: the promoter [{less than or equal to}1 kb from the transcription start site (TSS)] - composed of a core promoter 1, 2 and nearby (proximal) regulatory elements 3, 4, and more remote (distal) cis-regulatory elements ({greater than or equal to}1 kb from TSS), which can be enhancers, silencers, insulators, or locus control regions (LCRs) [3]" (Vernimmen and Bickmore, 2015; https://doi.org/10.1016/j.tig.2015.10.004). I share and have the impression that the predominant view in the field classifies enhancers as more distantly located to the TSS than the here presented "R7" sequence.

Suggested experiment Luciferase reporter assay R7 (timeline 1.5-3 months): To show that R7 acts as an enhancer, but not as a promoter, the authors should test activity of the element without the minimal promoter (if it retains significant activity, it can act as a promoter), and/or position the R7 element further away from the minimal promoter/in a different orientation, to see if it fits in with the classical definition of an enhancer.

Point 2: Comparison of WT and R7 deletion AC16 cells:
The experiments strongly support the conclusion that JAG1 is important for cardiomyocyte proliferation, migration, and survival

Point 3: Impact of R7 deletion on local chromatin topology:
The experiments supports the conclusion that R7-deletion alters the local chromatin 3D architecture at the JAG1 locus. This, however, does not mean that it is an enhancer

Point 4: Loss of R7 reduces local chromatin accessibility and affects heart-related biological processes: This statement is supported by the results.
There is literature evidence for promoter-anchored chromatin loops (Siersbaek et al., 2017; DOI: 10.1016/j.molcel.2017.04.010) and the observed changes in chromati could be a results of an enhancer deletion, but just as well stem from impairment of the promoter region of JAG1.

Point 5: Enhancer R7 coordinates transcription-associated chromatin features at the JAG1 locus
"..., was enriched at the R7 region and in the second intron under WT conditions, and this enrichment was significantly reduced in R7_KO cells, suggesting that deletion of R7 disrupts the activity of both the enhancer itself and a downstream regulatory region." - I do not understand this wording, of course if R7 is deleted, you will not find epigenomic marks at the R7 genomic region, as it is not present.
Again, the experiments make logical sense in the broader scope of the study, as part of a pipeline to regulate non-coding regulatory elements. However, I am still not convinvced R7 can be called an enhancer.

In this sections context, what is the authors opinion on R7 acting as an alternative promoter, considering distance to the TSS, high H3K4me3 occupancy (considered more of a promoter mark), and existence of this transcript (JAG1 isoform): Human Gene JAG1 (ENST00000901230.1) from GENCODE V49?

In conclusion, if the authors can make a convincint case for R7 not being a part of the promoter, I still have trouble with using just the term "enhancer" for its description. Maybe an alternative name can be found that this is not a "classical" enhancer, albeit R7 being a region important for efficient transcription.

Taken together, there is definitely an unmet clinical need for such data characterising the regulatory function of the non-coding genome, especially for genes with relevance in development and disease, as many patients remain without a molecular diagnosis. Therefore, the presented results are great proof of principle for the usefulness of the author's pipeline. It would have been interesting to see if knockout of R5 would have also resulted in a (similar) phenotype in the cellular model.
Given the authors computational expertise, checking for reported patient variants in the identified regulatory elements via R-panel could add additional clinical relevance to their approach.

3. Additional Issues
The authors should definitely be more clear about the short distance of the identified regulatory element to the TSS and this should be reflected in the title.

Text/format suggestions: To explore heart-associated enhancers surrounding the JAG1 locus, we employed a previously established tissue-specific enhancer prediction pipeline, R-panel. Please add citation nr. 25.

For clarity, in figure 5, I suggest different colors for the grey highlights, e.g. R7 could be highlighted in red to make it more obvious that this is the region absent in the KO line.

Some abbreviations are not defined in the text.

Response to the Editors and Reviewers

Dear Editors and Reviewers,

We sincerely thank the editors and reviewers for their careful evaluation of our manuscript and for their constructive comments. We appreciate the editor's recognition of our efforts to identify and characterize a regulatory element at the *JAG1* locus, and we also fully acknowledge the major concerns raised during review. In particular, the editorial decision highlighted the need to reconsider the designation of R7 as an enhancer, given its close proximity to the *JAG1* transcription start site, to provide additional functional evidence, to address the possibility that R7 may display promoter-like regulatory properties, and to discuss the limitations of the AC16 cell model and the lack of direct physiological validation in cardiac development.

In response, we have substantially revised the manuscript. We changed the title and terminology throughout the manuscript to describe R7 as a "promoter-proximal regulatory element with enhancer-like activity," rather than assigning it solely as an enhancer. We added new luciferase reporter assays using reverse-orientation and promoterless R7 constructs to evaluate the enhancer-like and promoter-like reporter properties of R7. We also performed *JAG1* restoration and overexpression experiments in R7-deletion AC16 cells to test whether reduced *JAG1* expression contributes to the observed cellular phenotypes. In addition, we revised the interpretation of the chromatin interaction, ATAC-seq, and ChIP-seq data to avoid overstatement, updated the Methods and figure legends, and expanded the Discussion to address the limitations of the current study.

Below, we provide a point-by-point response to the reviewers' comments. The reviewers' comments are reproduced in *italics*, and our responses are shown in blue text. Where appropriate, revised or newly added results are included below the corresponding responses for clarity.

Response to Reviewers

Reviewer #1 (Comments to the Authors)

In this report, cardiac-specific enhancers for JAG1 gene were investigated first through integrated analysis of the public genomic/epigenomic datasets, followed by reporter assay (positive) and deletion in AC16 cells. The transcriptional and cellular impact of enhancer deletion were evaluated in AC16 wildtype and deletion mutant cells. Additional impact on epigenetic features and chromatin architecture was evaluated by ChiP seq, ATAC-seq and HiC. Finally, a candidate transcription factor SRF was proposed. The authors concluded from these results that an SRF occupying enhancer for JAG1 was detected, implicating potential mechanism of SRF mediated regulation for JAG1 expression.

JAG1 is an important regulator in development with a major role in fetal cardiovascular differentiation and growth. Uncover the regulatory mechanism for JAG1 expression through enhancer identification would be valuable. However, there are major limitations in this study.

Response:

We thank the reviewer for the careful evaluation of our study and for recognizing the potential value of identifying regulatory mechanisms controlling *JAG1* expression. We also appreciate the reviewer's constructive comments regarding the limitations of the original manuscript. In the revised manuscript, we have made substantial changes to address these concerns. We revised the title and terminology to describe R7 more cautiously as a promoter-proximal regulatory element with enhancer-like activity, added new luciferase reporter assays to further characterize the regulatory properties of R7, performed *JAG1* restoration and overexpression experiments in R7-deletion AC16 cells, and revised the interpretation of the chromatin interaction, ATAC-seq, ChIP-seq, and SRF-related results to avoid overstatement. We have also expanded the Methods and Discussion sections to clarify the experimental design, data-analysis procedures, and limitations of the current study. Detailed responses to each point are provided below.

REVIEWER #1 POINT 1

-The R-pipeline used to identify the initial cardiac-specific enhancers for JAG1 should be carefully described in detail, with all the parameters and filtering criteria leading to the selection of the final 19 candidates.

Response:

We thank the reviewer for this helpful suggestion. We agree that the enhancer prediction and filtering strategy should be described in greater detail. In the revised manuscript, we have expanded the Methods section to provide a more detailed description of the R-panel pipeline, which was previously developed to prioritize heart-associated enhancer candidates by integrating tissue-enriched ChIP-seq signals(Wang F et al, 2022). We also clarified the filtering criteria used to select candidate heart-associated regulatory regions near the JAG1 locus.

Specifically, we now state that public ChIP-seq peak datasets for H3K4me1, H3K4me3, and H3K27ac from heart, liver, kidney, and brain tissues in human and mouse were obtained from GEO and ENCODE through Cistrome DB. Datasets with unhealthy or disease-related annotations were excluded, and only datasets passing the quality-control criteria of the original R-panel pipeline were retained. We further clarified that the retained datasets met Cistrome DB quality-control requirements, that peak coordinates were converted from hg38 or mm10 to hg19 using LiftOver, that mouse-to-human LiftOver used a minimum remapping ratio of 0.5, and that peaks with a length-change ratio greater than 1 after LiftOver were excluded.

We also clarified that the retained datasets were grouped by species and histone mark into six categories, and that heart-associated enrichment scores were calculated by comparing peak occurrence in heart samples with that in non-cardiac control tissues and normalized to an importance-score scale from 0 to 1,000. For the *JAG1* locus, candidate peaks within a ±100 kb window with scores ≥749 were retained, resulting in 19 candidate fragments. Adjacent retained peaks with an inter-peak distance <1,000 bp were then merged into R1–R10 candidate regions for luciferase-based functional screening.

Changes made:

We expanded the Methods section “Identification and clustering of candidate enhancer regions” to clarify the data sources, filtering criteria, scoring strategy, and peak-merging rules used to select the candidate regions.

REVIEWER #1 POINT 2

- Authors used AC16 cells to demonstrate tissue-specificity but failed to provide a non-myocyte cell line as a key control. The enhancer AC16 cells were extensively characterized, but the functional impact should be validated by restoring JAG1 expression through an ectopic expression vector. This is a very important control to support that all the observed phenotypic changes are indeed caused by the loss of JAG1 expression.

Response:

We thank the reviewer for this important comment. We agree that experiments performed in AC16 cells alone are not sufficient to establish tissue specificity. In our study, the initial prioritization of candidate regions was based on the R-panel screening strategy, which compares enhancer-associated epigenomic signals in heart tissues with those in non-cardiac tissues, including liver, kidney, and brain. Therefore, in the revised manuscript, we describe these regions as “heart-associated” or “cardiac-associated” candidate regulatory regions, rather than claiming tissue specificity based solely on AC16 cell experiments. We have also expanded the Discussion to acknowledge that R7 activity and function should be further compared across additional cardiac and non-cardiac contexts.

To address the reviewer’s request for functional validation, we performed new *JAG1* restoration and overexpression experiments in R7-deletion AC16 cells. R7_KO cells were transfected with different amounts of JAG1 expression plasmid. The condition that restored *JAG1* mRNA to a level comparable to WT cells was designated *JAG1*-RE, whereas the condition that resulted in *JAG1* expression above the WT level was designated *JAG1*-OE. We then examined cell-cycle progression, cell viability, and apoptosis in WT, R7_KO, *JAG1*-RE, and *JAG1*-OE cells.

These new experiments showed that both *JAG1*-RE and *JAG1*-OE reduced the G2 accumulation and apoptosis observed in R7_KO cells. In the CCK-8 assay, *JAG1*-RE produced a modest improvement in cell viability, whereas *JAG1*-OE showed a stronger trend toward restoration and approached the WT level at later time points. These results support the interpretation that reduced *JAG1* expression contributes to the cellular phenotypes caused by R7 deletion. We have described these effects as partial alleviation rather than complete rescue.

In the revised manuscript, these new *JAG1* restoration and overexpression data are presented in Figure 2. The previous EdU incorporation and wound-healing

assays performed in WT and R7_KO cells have been moved to Supplementary Figure S1 as supporting data. In addition, the apoptosis assay was performed using an updated Annexin V-FITC/PI apoptosis detection kit, and this change has been reflected in the revised Methods section.

For clarity, the revised Figure 2 is included below.

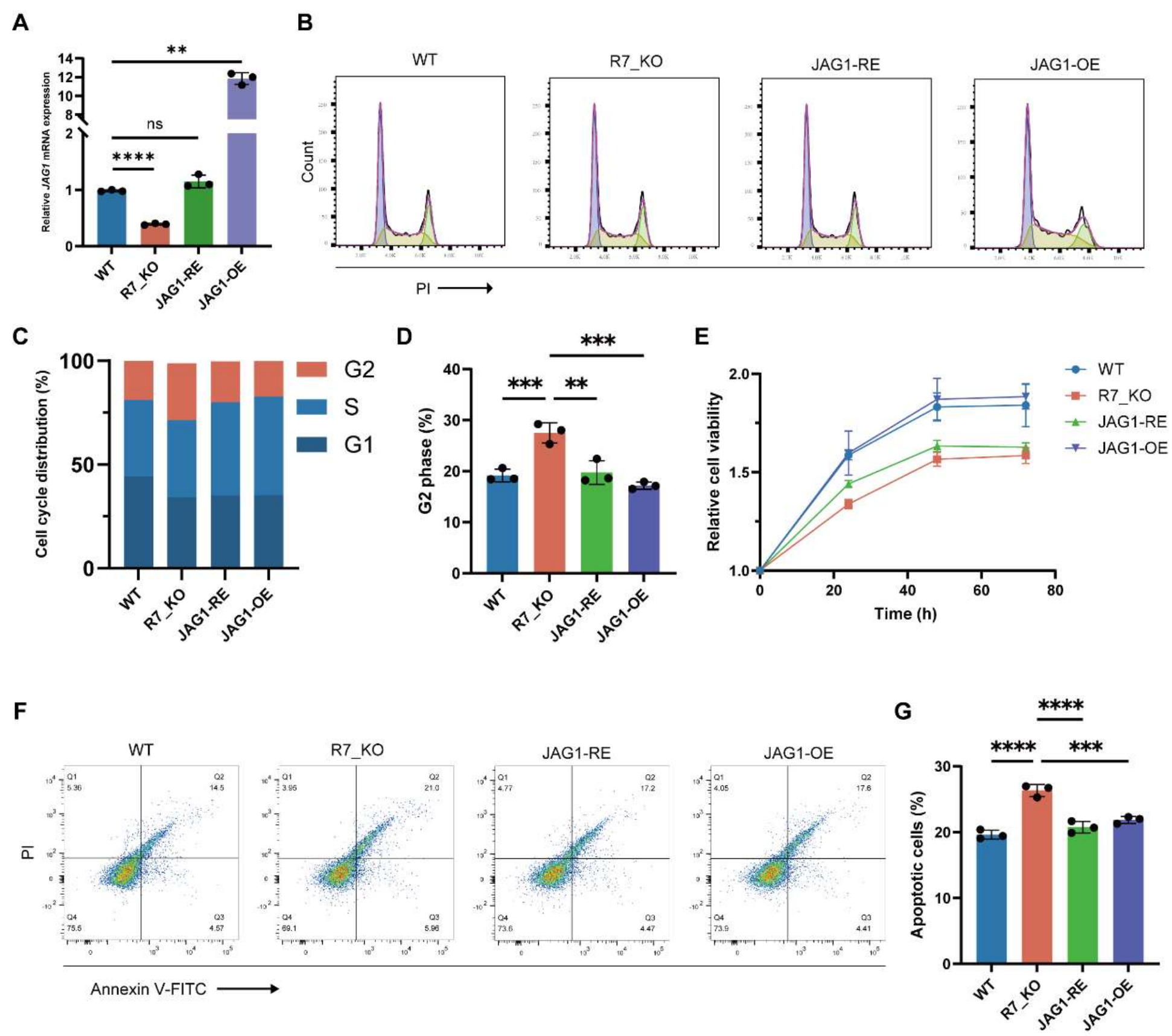

Figure 2. R7 deletion affects AC16 cell-cycle progression, cell viability, and apoptosis, with partial alleviation by *JAG1* restoration or overexpression.

(A) RT-qPCR analysis of *JAG1* mRNA expression in WT, R7_KO, *JAG1*-RE, and *JAG1*-OE cells. *JAG1*-RE indicates restoration of *JAG1* expression to near-WT levels, whereas *JAG1*-OE indicates higher *JAG1* overexpression in R7_KO cells. (B) Representative PI-staining flow-cytometry profiles for cell-cycle analysis. (C) Quantification of cell-cycle phase distribution. (D) Quantification of G2/M-phase cells. R7 deletion increased the G2/M population, which was reduced by *JAG1* restoration or overexpression. (E) CCK-8 assay showing relative cell viability over time. (F) Representative Annexin V-FITC/PI flow-cytometry plots for apoptosis analysis. (G) Quantification of apoptotic cells. Data are shown as mean ± SD. Statistical significance was determined by two-tailed unpaired Student's t-test for the indicated comparisons. **P < 0.01, ***P < 0.001, ****P < 0.0001; ns, not significant.

REVIEWER #1 POINT 3

- Without any in vivo demonstration of the enhancer activity or the epigenetic/chromatin features of the enhancer locus in heart, it is not clear about the physiological validity of the enhancer activity in the context of cardiac development or physiology. AC16 is not a fully differentiated myocyte model. This is another major limitation of this study.

Response:

We thank the reviewer for this important comment. We agree that AC16 cells are not a fully differentiated cardiomyocyte model and that direct validation in developmental or in vivo models is required to fully establish the physiological function of R7.

To provide additional support for the cardiac developmental context of the R7-containing region, we analyzed public cardiac developmental ChIP-seq datasets, including mouse cardiac p300 ChIP-seq data (GSE195901) (Zhou P et al, 2023) and H3K27ac/CTCF ChIP-seq data from human iPSC-derived cardiomyocyte differentiation (GSE116862) (Zhang Y et al, 2019). These datasets showed regulatory chromatin signals at the mouse *Jag1* / human *JAG1* regulatory region, including H3K27ac/CTCF enrichment at the human R7-containing promoter-proximal region. These data have been added as Supplementary Figure S3.

We have also revised the Discussion to acknowledge that these public epigenomic data provide supportive developmental context but do not replace direct functional validation. We now state that the physiological function of R7 should be further tested in developmental and in vivo models, as well as in additional cardiac and non-cardiac cellular contexts.

For clarity, the newly added Supplementary Figure S3 is shown below.

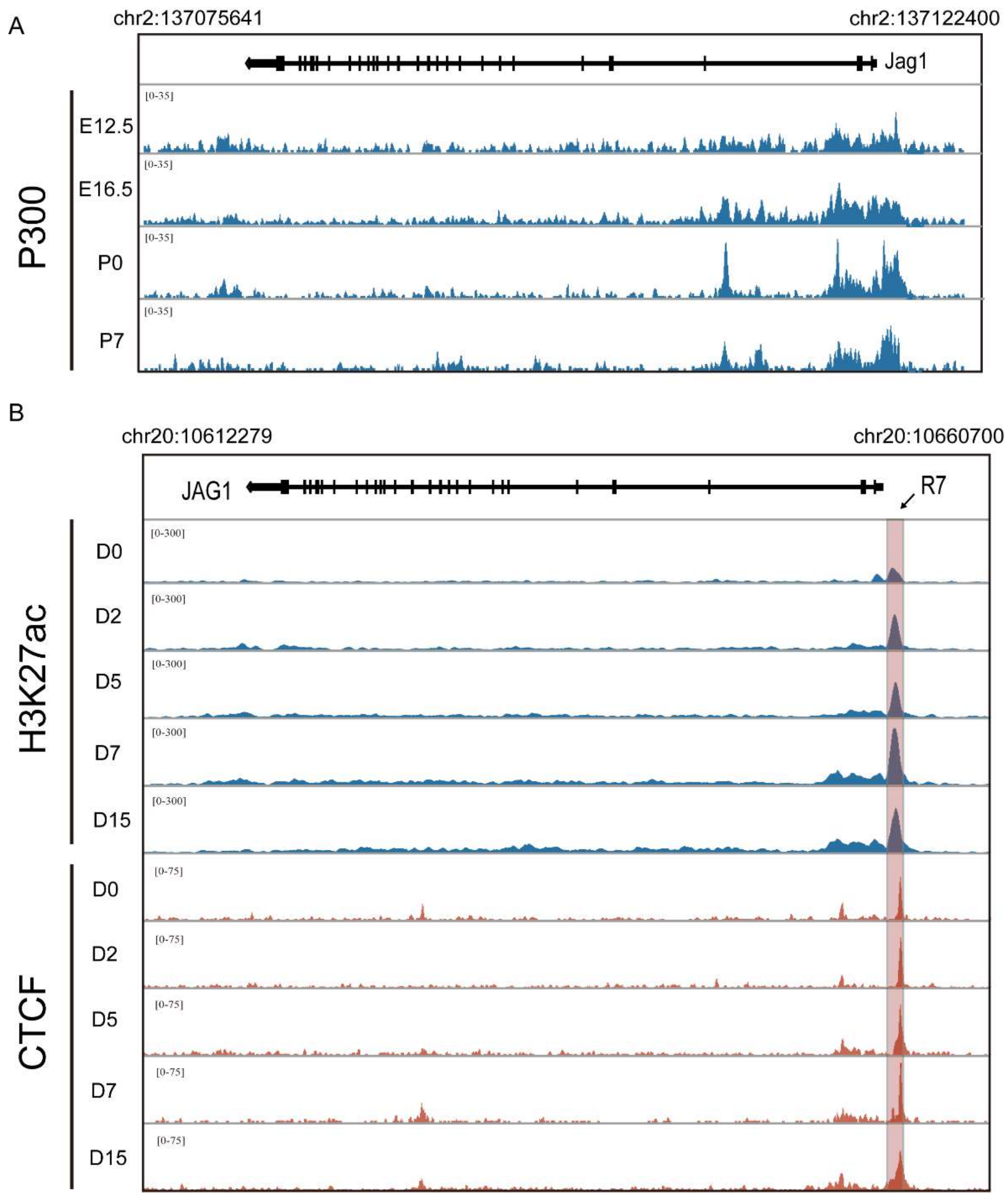

Supplementary Figure S3. Public cardiac developmental ChIP-seq datasets show regulatory chromatin signals near the mouse *Jag1* / human *JAG1* locus.

(A) Public p300 ChIP-seq profiles across the mouse Jag1 locus at indicated cardiac developmental stages (GSE195901). E indicates embryonic day, and P indicates postnatal day. (B) Public H3K27ac and CTCF ChIP-seq profiles across the human JAG1 locus during iPSC-derived cardiomyocyte differentiation (GSE116862). D indicates differentiation day after initiation of cardiac differentiation. The shaded region indicates the R7-containing promoter-proximal region.

Changes made:

We added public cardiac developmental ChIP-seq analyses as Supplementary Figure S3, described these data in Results section 5, updated the Data availability statement to include GSE195901 and GSE116862, and expanded the Discussion to acknowledge the need for further developmental and in vivo validation.

REVIEWER #1 POINT 4

-The authors presented only correlative and descriptive data on the SRF as a candidate trans-acting TF for JAG1 activity. Its contribution to local enhancer activity, chromatin and epigenetic reprogramming should be direct tested in Wildtype vs. KO cells using targeted KD or Over-expression approaches.

Response:

We thank the reviewer for this important comment. We agree that the current SRF data are correlative and do not establish a direct causal role for SRF in mediating the regulatory effects of R7.

In the original analysis, SRF was selected as a candidate transcription factor based on a published murine cardiac transcription factor bioChIP-seq dataset GSE124008 (Akerberg BN et al, 2019). Specifically, we used the ATAC-seq open chromatin peaks at R7 and the *JAG1* second-intron region, lifted these regions over to the mouse genome, and examined transcription factor occupancy at the corresponding murine cardiac regulatory loci. SRF occupancy was observed in this public dataset and was therefore selected for further ChIP-seq analysis in AC16 cells.

In the revised manuscript, we have weakened the interpretation of SRF-related findings. We now describe SRF as a candidate factor associated with the R7-linked regulatory region, rather than as a proven mediator of R7 function. We also clarified that our ChIP-seq data show reduced SRF occupancy across the R7-associated promoter-proximal and intronic regulatory region after R7 deletion, but do not demonstrate that SRF directly drives the chromatin or transcriptional effects observed after R7 deletion. We have revised the Discussion to state that the causal contribution of SRF remains to be tested in future perturbation-based experiments, such as targeted knockdown or

overexpression.

Changes made:

We revised the Candidate TF Identification section to clarify how SRF was selected from the published murine cardiac TF bioChIP-seq dataset (GSE124008; Akerberg et al., 2019). We also revised Results section 5 and the Discussion to describe SRF as an associated candidate factor and to acknowledge that its causal role requires future perturbation-based validation.

REVIEWER #1 POINT 5

-The authors presented only one AC16 enhancer KO cell line. Considering the potential off-target effects from Crispr based genome editing, it is important to show at least an alternative cell line for functional validation.

Response:

We agree that using a single genome-edited AC16 clone is a limitation and that validation in additional independently edited clones or cellular models would further strengthen the conclusion. To partially address this concern and provide an orthogonal functional test, we performed new JAG1 restoration and overexpression experiments in R7_KO cells.

These experiments showed that restoration of *JAG1* expression partially alleviated the cell-cycle and apoptosis phenotypes caused by R7 deletion, supporting the interpretation that the observed cellular defects are linked, at least in part, to reduced *JAG1* expression. However, we agree that these experiments do not fully exclude clone-specific or CRISPR-related effects. We have therefore explicitly acknowledged this limitation in the Discussion and now state that future studies should test R7 activity and function in independent genome-edited clones and additional cardiac and non-cardiac cellular contexts, including primary cardiomyocytes and iPSC-derived cardiomyocytes.

Changes made:

We added *JAG1* restoration and overexpression experiments in Figure 2 and expanded the Discussion to acknowledge that potential clone-specific or CRISPR-related effects cannot be fully excluded and that future validation in

independent genome-edited clones and additional cellular models is needed.

REVIEWER #1 POINT 6

- All bar-graph results should be presented with all data points in order to illustrate reproducibility of the data. Similar concerns about the epigenetic, chromatin profiling. It is unclear how many samples were analyzed and whether the differences illustrated were reproducible.

Response:

We thank the reviewer for this helpful suggestion. In the revised figures, we have shown individual data points in the bar graphs where applicable, together with mean ± SD, to better illustrate reproducibility. The functional assays presented in bar graphs were performed using three biological replicates.

We also clarified the replicate information for the sequencing-based chromatin and epigenomic profiling experiments in the revised Methods. Specifically, 4C-seq was performed using two biological replicates for each genotype, ATAC-seq was performed using two biological replicates for each group, and ChIP-seq was performed using two biological replicates for each condition and target. In addition, all sequencing data generated in this study, including RNA-seq, ATAC-seq, ChIP-seq, and 4C-seq datasets, have been deposited in the European Nucleotide Archive under accession number PRJEB102413 (ERP183811).

Changes made:

We revised the bar graphs to show individual data points where applicable, clarified that functional assays were performed with three biological replicates, added replicate information for 4C-seq, ATAC-seq, and ChIP-seq in the Methods, and updated the Data availability statement for the sequencing datasets.

Summary and final appreciation for Reviewer #1

We sincerely thank Reviewer #1 for the thorough evaluation and constructive suggestions. The reviewer's comments were highly valuable in helping us strengthen both the experimental support and the clarity of interpretation in the revised manuscript. In response, we expanded the description of the R-panel screening strategy and provided additional details on data sources, filtering criteria, scoring methods, and candidate-region selection. We also added *JAG1* restoration and overexpression experiments in R7-deletion AC16 cells, which provided important functional evidence that reduced *JAG1* expression contributes to the cellular phenotypes observed after R7 deletion.

The reviewer's comments also helped us better define the limitations and appropriate interpretation of the study. We added public cardiac developmental epigenomic datasets to provide additional regulatory context, while clearly acknowledging that these data do not replace direct in vivo validation. We also revised the SRF-related conclusions to avoid implying a causal role without perturbation-based evidence, clarified the limitation of using a single genome-edited AC16 clone, and added replicate information for sequencing-based chromatin and epigenomic profiling experiments. In addition, we revised the bar graphs to show individual data points where applicable, improving the transparency of data presentation.

Together with the comments from the editors and Reviewer #3, Reviewer #1's suggestions were instrumental in guiding us to present the study in a more rigorous and balanced manner. We now describe R7 as a promoter-proximal regulatory element with enhancer-like activity, rather than assigning it solely as an enhancer, and we have revised the manuscript to more carefully distinguish functional evidence, associated chromatin features, and remaining limitations. We believe these revisions have substantially improved the rigor, transparency, and overall strength of the manuscript, and we sincerely appreciate the reviewer's help.

REVIEWER #3 Summary of the paper

- Non-coding patient variants mapping to, for example, enhancers, are likely to contribute to a significant amount of (congenital) disease cases, but causal patient variants are harder to identify due to a knowledge gap in the understanding of the cis-regulatory mechanisms regulating gene expression. The authors identify a relevant and interesting challenge in the field: How to best systematically screen for and prioritize enhancer candidates, and how to then functionally test them in order to identify the key regulatory elements that govern tissue-specific transcriptional regulation. After prioritizing enhancer candidates, they use a reporter assay for functional evaluation. From the 2 identified active regions, they chose to delete the strongest element in a human cell line model of cardiomyocytes and characterize the chromatin state, histone modifications, and 3D chromatin structure, identifying changes at the JAG1 locus and on a genome-wide level. They also observe cellular phenotypes that are in line with impaired ventricular development in a murine JAG1 knockout model. The paper presents a comprehensive pipeline that spans enhancer prediction, epi-/genomic characterisation and functional testing, spanning the key steps to characterise regulatory elements and to prioritize genomic regions relevant to diagnose and potentially treat congenital heart disease.

Response:

We appreciate the reviewer's thoughtful summary of our study and the recognition of the broader importance of systematically identifying and functionally characterizing noncoding regulatory elements relevant to congenital heart disease. We agree that a better understanding of cis-regulatory mechanisms at disease-relevant loci is important for improving the interpretation of noncoding variants.

In revising the manuscript, we have taken the reviewer's overall assessment and the specific concerns raised below into careful consideration. In particular, we have refined the terminology and interpretation of R7 to better reflect its genomic context. Because R7 is located close to the *JAG1* transcription start site, we now describe it as a cardiac-associated promoter-proximal regulatory element with enhancer-like activity, rather than assigning it solely as a classical enhancer. We also added new reporter assays and *JAG1* restoration/overexpression experiments to strengthen the functional interpretation of the R7 element, while revising the chromatin-related conclusions to avoid overstatement.

REVIEWER #3 POINT 1

-Point 1: Enhancer prediction pipeline and functional validation/prioritization using a dual-luciferase reporter assay.

The authors used their "previously established" R-panel to identify enhancers based on an integrated analysis pipeline of 148 ChIP-seq datasets from several tissues, including heart. Ten enhancers/enhancer clusters were identified for testing in a reporter assay and 2 sequences showed significantly stronger luciferase signal, indicating their ability to enhance target gene transcription. This is a solid strategy to efficiently screen a large genomic locus (+/- 100kb window). They then chose to continue with the detailed characterisation of "R7", which is located 253bp upstream of the JAG1 TSS.

The CRISPR/Cas9-deletion of R7 seems to have been introduced by cutting even closer to the TSS than 253bps.

Major criticism: Typically, enhancers act at a distance of few kilobases to tens of kilobases (Gasperini, M., Tome, J.M. & Shendure, J. Towards a comprehensive catalogue of validated and target-linked human enhancers. Nat Rev Genet 21, 292-310 (2020). https://doi.org/10.1038/s41576-019-0209-0). This review, for example, gives a cutoff of a distance of over 1000bp from the promoter for enhancers. See: "Genes transcribed by RNA polymerase (Pol)II usually have two distinct families of cis-acting elements: the promoter [less than or equal to1 kb from the transcription start site (TSS)] - composed of a core promoter 1, 2 and nearby (proximal) regulatory elements 3, 4, and more remote (distal) cis-regulatory elements (greater than or equal to1 kb from TSS), which can be enhancers, silencers, insulators, or locus control regions (LCRs) [3]" (Vernimmen and Bickmore, 2015; https://doi.org/10.1016/j.tig.2015.10.004). I share and have the impression that the predominant view in the field classifies enhancers as more distantly located to the TSS than the here presented "R7" sequence.

Suggested experiment Luciferase reporter assay R7 (timeline 1.5-3 months): To show that R7 acts as an enhancer, but not as a promoter, the authors should test activity of the element without the minimal promoter (if it retains significant activity, it can act as a promoter), and/or position the R7 element further away from the minimal promoter/in a different orientation, to see if it fits in with the classical definition of an enhancer.

Response:

This is an important and very helpful point. We agree with the reviewer that our original designation of R7 as an enhancer did not sufficiently take into account its close proximity to the *JAG1* transcription start site. R7 was initially selected for further characterization because it was identified by our enhancer-associated R-panel screening strategy and showed the strongest reporter activity among the tested candidate regions. However, as the reviewer correctly points out, R7 is located only 253 bp upstream of the JAG1 TSS, placing it within a promoter-proximal genomic context. We therefore agree that it would be inappropriate to define R7 solely as a classical enhancer. In revising the manuscript, we followed the current practice of interpreting candidate cis-regulatory elements by considering not only reporter activity, but also endogenous genomic context and target-gene linkage(Gasperini M et al, 2020). Because promoter-proximal sequences can also display regulatory properties that overlap with enhancer function(Dao LTM et al, 2017; Vernimmen D & Bickmore WA, 2015), we now describe R7 as a cardiac-associated promoter-proximal regulatory element with enhancer-like activity. We also appreciate the reviewer's careful observation that the CRISPR/Cas9 deletion was introduced by cutting even closer to the TSS; we have now clarified in the revised Methods and Figure 1F legend that the proximal sgRNA cut site was located approximately 228 bp upstream of the JAG1 TSS.

Following the reviewer's suggestion, we generated two additional luciferase reporter constructs. First, R7 was inserted upstream of the E1b minimal promoter in the reverse orientation. The R7_Reverse construct retained reporter activity, supporting enhancer-like reporter activity. Second, we removed the E1b minimal promoter and placed R7 directly upstream of the luciferase coding sequence. The R7_ΔE1b construct showed activity comparable to the forward R7-E1b construct, indicating promoter-like reporter activity in this assay.

These new results support a revised interpretation that R7 has both enhancer-like and promoter-like reporter properties. This interpretation is consistent with the concept that promoter-associated regulatory sequences may exhibit enhancer-like activity in reporter or endogenous genomic contexts(Dao LTM & Spicuglia S, 2018). We therefore revised the title, terminology, Results, Discussion, Methods, and figure legends accordingly. We appreciate the reviewer's suggestion, which helped us make the definition and interpretation of R7 more accurate.

For clarity, the revised Figure 1 is shown below.

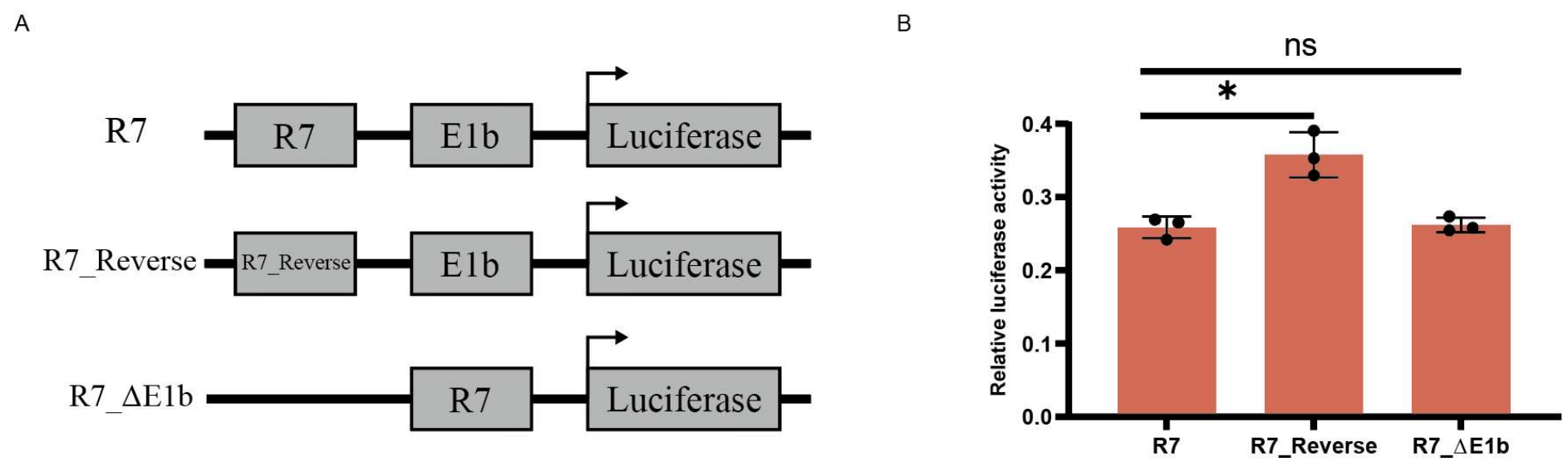

Figure1. Reporter analysis of R7 orientation-dependent and promoterless activity.

(A) Schematic of R7, R7_Reverse, and R7_ΔE1b luciferase reporter constructs.

(B) Relative luciferase activity of the indicated constructs in AC16 cells. Data are shown as mean ± SD. Statistical significance was determined by two-tailed unpaired Student's t-test. *P < 0.05; ns, not significant.

Changes made:

We changed the title to "*A Cardiac-Associated Promoter-Proximal Regulatory Element Shapes JAG1 Chromatin and Transcription*" We revised the terminology throughout the manuscript, added the R7_Reverse and R7_ΔE1b luciferase assays to Figure 1D–E and Result 1, updated the Methods to clarify the proximal sgRNA cut site relative to the *JAG1* TSS, and expanded the Discussion to address the promoter-proximal location and reporter properties of R7.

REVIEWER #3 POINT 2

- Point 2: Comparison of WT and R7 deletion AC16 cells:

The experiments strongly support the conclusion that JAG1 is important for cardiomyocyte proliferation, migration, and survival.

Response:

We appreciate the reviewer's positive assessment of these functional data. To further strengthen this conclusion, we added *JAG1* restoration and overexpression experiments in R7-deletion AC16 cells to test whether reduced *JAG1* expression contributes to the cellular defects observed after R7 deletion.

In the revised manuscript, restoration of *JAG1* expression to near-WT levels partially alleviated the G2/M accumulation and increased apoptosis in R7_KO cells. *JAG1* restoration also modestly improved cell viability, whereas *JAG1* overexpression showed a stronger trend toward recovery at later time points. These results support the interpretation that reduced *JAG1* expression contributes to the cellular phenotypes caused by R7 deletion, although the rescue was partial rather than complete. We have revised the wording accordingly to avoid overstatement.

Changes made:

We added *JAG1* restoration and overexpression experiments to revised Figure 2 and Result 2. The original EdU incorporation and wound-healing assays were moved to Supplementary Figure S1. We also revised the text to describe the effects as partial alleviation of R7-deletion-associated defects in AC16 cell-cycle progression, viability, and apoptosis.

REVIEWER #3 POINT 3

- Point 3: Impact of R7 deletion on local chromatin topology:

The experiments support the conclusion that R7-deletion alters the local chromatin 3D architecture at the JAG1 locus. This, however, does not mean that it is an enhancer.

Response:

We agree with the reviewer's interpretation. The chromatin interaction data show that deletion of R7 alters local chromatin contacts around the *JAG1* locus, but these results alone do not establish R7 as a classical enhancer. In the revised manuscript, we have therefore avoided using the chromatin topology data as evidence that R7 is an enhancer.

Accordingly, we revised this section to describe the result more conservatively. The section title was changed from "Enhancer R7 is essential for maintaining chromatin topology surrounding the *JAG1* locus" to "R7 deletion alters local chromatin interactions surrounding the *JAG1* locus." We also revised the Results and figure legend to state that R7 deletion reduces local contact signals and perturbs the 3D regulatory architecture surrounding the *JAG1* locus, without implying that this finding by itself defines R7 as an enhancer.

Changes made:

We revised Result 3, Figure 3 title, and Figure 3 legend to use more cautious wording. In particular, we removed wording that described R7 as an enhancer or as essential for chromatin topology, and instead described the data as showing altered local chromatin contacts after R7 deletion.

REVIEWER #3 POINT 4

Point 4: Loss of R7 reduces local chromatin accessibility and affects heart-related biological processes: This statement is supported by the results. *There is literature evidence for promoter-anchored chromatin loops (Siersbaek et al., 2017; DOI: 10.1016/j.molcel.2017.04.010) and the observed changes in chromatin features could be a result of an enhancer deletion, but just as well stem from impairment of the promoter region of JAG1.*

Response:

This is a thoughtful point, and we agree that the chromatin accessibility data cannot distinguish by themselves whether the observed changes result from loss of enhancer-like activity or from impairment of the *JAG1* promoter-proximal region. Our initial interpretation of R7 as an enhancer-associated regulatory region was based on its identification by the R-panel screening strategy, its reporter activity, and the local enrichment of ATAC-seq and CTCF signals at the R7-containing region and the *JAG1* second intron, rather than broadly across the surrounding 1-Mb region. These features suggested that R7 is part of a local regulatory region with chromatin accessibility and architectural features.

However, we agree with the reviewer that, given the close proximity of R7 to the *JAG1* TSS, these data should not be interpreted as evidence for a classical enhancer. In the revised manuscript, we therefore describe the affected region as the R7-containing promoter-proximal region and interpret the ATAC-seq changes as disruption of a local promoter-proximal regulatory environment. We also revised the Discussion to state that the observed chromatin changes may reflect impairment of promoter-proximal regulation, rather than loss of a classical enhancer alone, in line with the reviewer's point that promoter-anchored chromatin loops can participate in transcriptional regulation(Siersbaek R et al, 2017).

Changes made:

We revised Result 4 and the corresponding figure legend to avoid enhancer-specific interpretation of the ATAC-seq changes. We now describe R7 deletion as reducing chromatin accessibility around the R7-containing promoter-proximal region and the *JAG1* second intron. We also expanded the Discussion to include promoter-anchored chromatin loops and to emphasize that the chromatin changes may arise from perturbation of promoter-proximal regulation at the *JAG1* locus

REVIEWER #3 POINT 5

- Point 5: Enhancer R7 coordinates transcription-associated chromatin features at the JAG1 locus

"..., was enriched at the R7 region and in the second intron under WT conditions, and this enrichment was significantly reduced in R7_KO cells, suggesting that deletion of R7 disrupts the activity of both the enhancer itself and a downstream regulatory region." - I do not understand this wording, of course if R7 is deleted, you will not find epigenomic marks at the R7 genomic region, as it is not present.

Again, the experiments make logical sense in the broader scope of the study, as part of a pipeline to regulate non-coding regulatory elements. However, I am still not convinced R7 can be called an enhancer.

In this sections context, what is the authors opinion on R7 acting as an alternative promoter, considering distance to the TSS, high H3K4me3 occupancy (considered more of a promoter mark), and existence of this transcript (JAG1 isoform): Human Gene JAG1 (ENST00000901230.1) from GENCODE V49?

Response:

This is an important point, and we agree that our original wording was not sufficiently precise. We did not intend to suggest that epigenomic marks could still be detected at the deleted R7 sequence itself in R7_KO cells. Rather, our intended point was that, in WT cells, transcription-associated chromatin signals formed broader enrichment around the R7-containing promoter-proximal region and adjacent sequences, and that after R7 deletion, reduced signal was also observed in the remaining flanking promoter-proximal region and the *JAG1* second intron. We have revised the text to clarify this interpretation and to avoid implying that loss of signal at the deleted interval itself reflects reduced activity of the deleted sequence.

We also agree that R7 should not be described simply as an enhancer. As described above, we have revised the terminology throughout the manuscript and now refer to R7 as a promoter-proximal regulatory element with enhancer-like activity. Regarding the possibility that R7 may act as an alternative promoter, we agree that this possibility cannot be excluded. The close proximity of R7 to the *JAG1* TSS, the H3K4me3 signal around this region, the promoterless reporter activity of R7_ΔE1b, and the transcript annotation noted by the

reviewer are all consistent with promoter-like properties. At the same time, the retained reporter activity of R7_Reverse supports enhancer-like reporter activity. We therefore interpret R7 as having both enhancer-like and promoter-like reporter properties, while acknowledging that endogenous alternative-promoter activity at the *JAG1* locus remains to be tested in future studies.

Changes made:

We revised Result 5 and the Figure 5 legend to clarify that the observed reductions in chromatin signals refer to the broader flanking promoter-proximal region and the remaining promoter–intron regulatory region, rather than to the deleted R7 sequence itself. We changed the terminology throughout the manuscript from "enhancer R7" to "R7" or "promoter-proximal regulatory element with enhancer-like activity." We also revised the Discussion to state that R7 displays both enhancer-like and promoter-like reporter properties and that possible promoter-like or alternative-promoter-like function at the endogenous *JAG1* locus requires further investigation.

REVIEWER #3 Conclusion

- ***In conclusion, if the authors can make a convincing case for R7 not being a part of the promoter, I still have trouble with using just the term "enhancer" for its description. Maybe an alternative name can be found that this is not a "classical" enhancer, albeit R7 being a region important for efficient transcription.***

Taken together, there is definitely an unmet clinical need for such data characterising the regulatory function of the non-coding genome, especially for genes with relevance in development and disease, as many patients remain without a molecular diagnosis. Therefore, the presented results are great proof of principle for the usefulness of the author's pipeline. It would have been interesting to see if knockout of R5 would have also resulted in a (similar) phenotype in the cellular model. Given the authors computational expertise, checking for reported patient variants in the identified regulatory elements via R-panel could add additional clinical relevance to their approach.

Response:

We agree with the reviewer's overall conclusion. Given the close proximity of R7 to the *JAG1* TSS and its promoter-like reporter activity,

we cannot exclude the possibility that R7 contributes to promoter-proximal or promoter-like regulation. Therefore, we have adopted an alternative terminology and no longer describe R7 simply as an enhancer. Throughout the revised manuscript, we now refer to R7 as a promoter-proximal regulatory element with enhancer-like activity, which better reflects both its reporter activity and its genomic context.

We also appreciate the reviewer's positive assessment of the potential value of our pipeline for characterizing noncoding regulatory regions. We agree that functional analysis of R5 would be informative, particularly because R5 also showed reporter activity in the initial screen. In this revision, we focused on clarifying the regulatory identity and functional role of R7, but we now mention R5 as an additional candidate region that warrants future perturbation-based validation. We also agree that integrating reported patient variants with R-panel-defined regulatory intervals could further improve the clinical relevance of this approach. This will be an important focus of our future work, and we have added this point to the revised Discussion.

Changes made:

We revised the title and terminology throughout the manuscript to describe R7 as a promoter-proximal regulatory element with enhancer-like activity rather than as an enhancer. We also revised the Discussion to mention R5 as an additional candidate regulatory region for future functional testing and to note that integration of patient noncoding variants with R-panel-defined regulatory intervals may further support variant prioritization at the *JAG1* locus.

REVIEWER #3 Additional Issues

The authors should definitely be more clear about the short distance of the identified regulatory element to the TSS and this should be reflected in the title.

Text/format suggestions: To explore heart-associated enhancers surrounding the JAG1 locus, we employed a previously established tissue-specific enhancer prediction pipeline, R-panel. Please add citation nr. 25.

For clarity, in figure 5, I suggest different colors for the grey highlights, e.g. R7 could be highlighted in red to make it more obvious that this is the region absent in the KO line. Some abbreviations are not defined in the text.

Response:

We appreciate these helpful suggestions. We have revised the title to explicitly reflect the promoter-proximal location of R7 and now refer to it as a “promoter-proximal regulatory element.” This change is also consistent with our revised interpretation of R7 throughout the manuscript.

We have also added the citation for the previously established R-panel method at the first mention of the pipeline. In addition, we revised Figure 5 to distinguish the R7 interval more clearly from the surrounding highlighted regions, with the R7-deleted region indicated in red. Finally, we reviewed the manuscript and defined abbreviations at first use where needed.

For clarity, the revised Figure 5 is shown below.

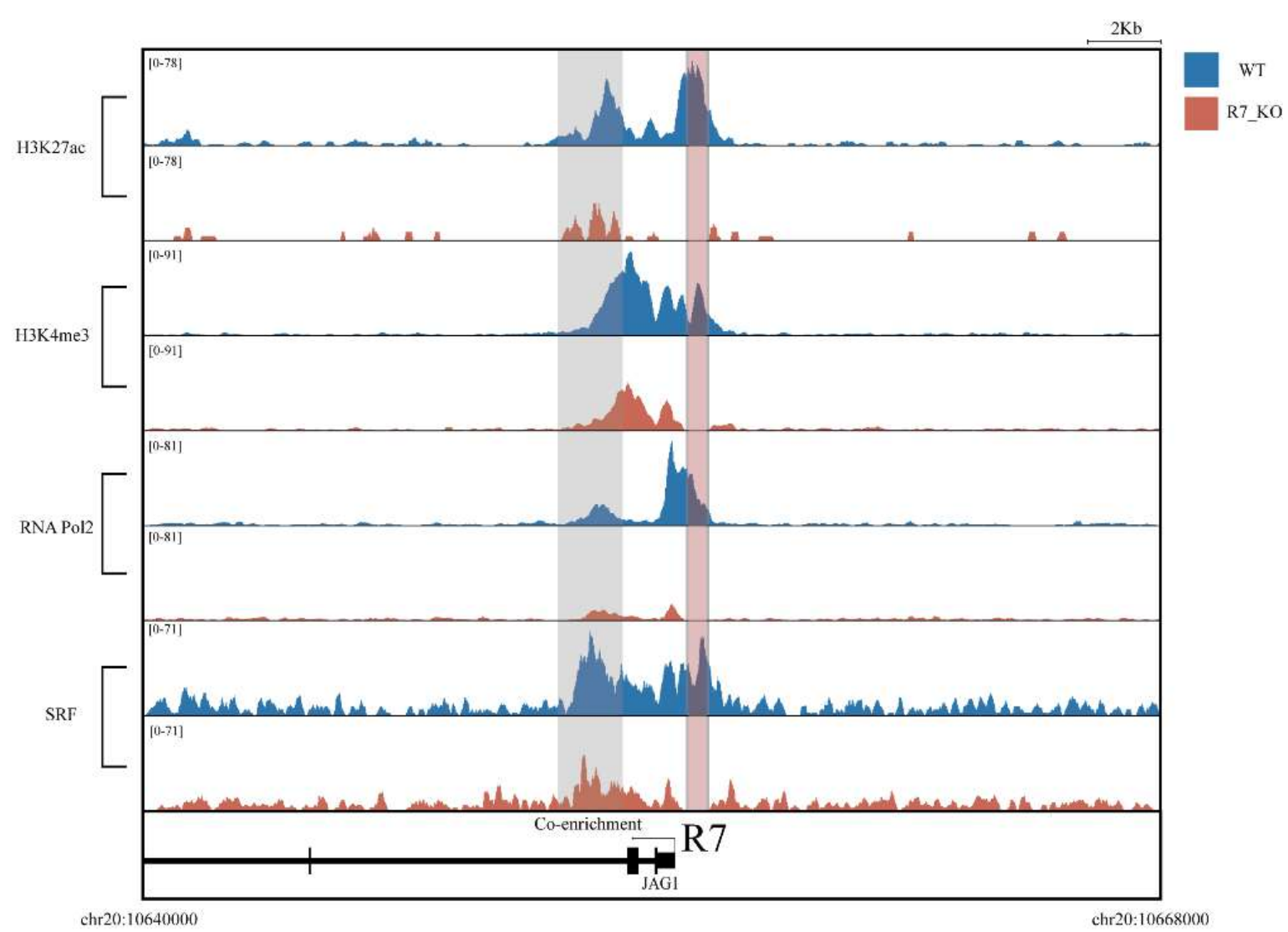

Changes made:

We revised the manuscript title, added the citation for the R-panel method, updated Figure 5 to highlight the R7-deleted interval more clearly, and defined previously undefined abbreviations in the text.

Summary and final appreciation for Reviewer #3

We sincerely thank Reviewer #3 for the thoughtful evaluation and constructive suggestions. The reviewer's comments were particularly helpful in guiding us to reconsider the regulatory identity of R7 in the context of its close proximity to the *JAG1* TSS. Together with the comments from Reviewer #1 and the editorial assessment, these suggestions prompted us to revise the title and terminology throughout the manuscript and to describe R7 more accurately as a promoter-proximal regulatory element with enhancer-like activity, rather than simply as an enhancer.

Reviewer #3's suggestions also directly motivated the additional luciferase reporter assays using reverse-orientation and promoterless R7 constructs. These experiments clarified that R7 displays both enhancer-like and promoter-like reporter properties. In addition, the reviewer's comments helped us revise the interpretation of the chromatin interaction, ATAC-seq, and ChIP-seq results to avoid overstatement and to distinguish more clearly between enhancer-like activity, promoter-proximal regulation, and possible promoter-like or alternative-promoter-like function.

We also appreciate the reviewer's broader comments on the value of systematic noncoding regulatory-element screening. In response, we added discussion of R5 as an additional candidate regulatory region for future functional testing and noted that integrating patient noncoding variants with R-panel-defined regulatory intervals will be an important future direction. We believe that these revisions, together with the additional functional evidence added in response to Reviewer #1, have substantially improved the precision, balance, and translational relevance of the manuscript.

References

Akerberg BN, Gu F, VanDusen NJ, Zhang X, Dong R, Li K, Zhang B, Zhou B, Sethi I, Ma Q, et al (2019) A reference map of murine cardiac transcription factor chromatin occupancy identifies dynamic and conserved enhancers. *Nat Commun* 10: 4907. doi:10.1038/s41467-019-12812-3

Dao LTM, Galindo-Albarran AO, Castro-Mondragon JA, Andrieu-Soler C, Medina-Rivera A, Souaid C, Charbonnier G, Griffon A, Vanhille L, Stephen T, et al (2017) Genome-wide characterization of mammalian promoters with distal enhancer functions. *Nat Genet* 49: 1073–1081. doi:10.1038/ng.3884

Dao LTM, Spicuglia S (2018) Transcriptional regulation by promoters with enhancer function. *Transcription* 9: 307–314. doi:10.1080/21541264.2018.1486150

Gasperini M, Tome JM, Shendure J (2020) Towards a comprehensive catalogue of validated and target-linked human enhancers. *Nat Rev Genet* 21: 292–310. doi:10.1038/s41576-019-0209-0

Siersbaek R, Madsen JGS, Javierre BM, Nielsen R, Bagge EK, Cairns J, Wingett SW, Traynor S, Spivakov M, Fraser P, et al (2017) Dynamic rewiring of promoter-anchored chromatin loops during adipocyte differentiation. *Mol Cell* 66: 420–435 e425. doi:10.1016/j.molcel.2017.04.010

Vernimmen D, Bickmore WA (2015) The hierarchy of transcriptional activation: From enhancer to promoter. *Trends in Genetics* 31: 696–708. doi:10.1016/j.tig.2015.10.004

Wang F, Zhang Y, Wu F, Gui Y, Chen X, Wang Y, Wang X, Gui Y, Li Q (2022) Functional assessment of heart-specific enhancers by integrating chip-seq data. *Pediatr Res* 92: 1332–1340. doi:10.1038/s41390-022-01981-5

Zhang Y, Li T, Preissl S, Amaral ML, Grinstein JD, Farah EN, Destici E, Qiu Y, Hu R, Lee AY, et al (2019) Transcriptionally active herv-h retrotransposons demarcate topologically associating domains in human pluripotent stem cells. *Nat Genet* 51: 1380–1388. doi:10.1038/s41588-019-0479-7

Zhou P, VanDusen NJ, Zhang Y, Cao Y, Sethi I, Hu R, Zhang S, Wang G, Ye L, Mazumdar N, et al (2023) Dynamic changes in p300 enhancers and enhancer-promoter contacts control mouse cardiomyocyte maturation. *Dev Cell* 58: 898–914 e897. doi:10.1016/j.devcel.2023.03.020

June 22, 2026

RE: Life Science Alliance Manuscript #LSA-2025-03597-TR

Prof. Guoying Huang
Children's Hospital of Fudan University
No. 399 Wanyuan Road
Shanghai 201102
China

Dear Dr. Huang,

Thank you for submitting your revised manuscript entitled "A Cardiac-Associated Promoter-Proximal Regulatory Element Shapes JAG1 Chromatin and Transcription". Your revised manuscript has been evaluated by one of the original reviewers, whose comments are appended below. As you will read, this reviewer acknowledged the changes to your revised version and the improvement. In line with the reviewer's evaluation, we would be happy to publish your paper in Life Science Alliance pending final revisions necessary to meet our formatting guidelines.

MANUSCRIPT ORGANIZATION AND FORMATTING:

To avoid unnecessary delays in the acceptance and publication of your paper, please read the following information carefully. Full guidelines are available on our Instructions for Authors page, https://www.life-science-alliance.org/authors

Submission of a paper that does not conform to Life Science Alliance guidelines will delay the acceptance of your manuscript.

-We suggest you to change your title to, 'A cardiac-related, promoter-proximal, regulatory element shapes chromatin and JAG1 transcription'.
-Kindly include molecular weight markers for blots shown in Figures 1J, Axis titles for Figure 4B, S2 scale bars for Figure S1.
-Please upload your Tables in editable .doc or Excel format; They can be included at the bottom of the main manuscript file or sent as separate files.
-In your methods, please cite the ChIPSeq peak datasets you mention in the section on identification of enhancer regions.
-Kindly expand on the imaging for cell migration assays with details on objectives and image acquisition.
-Please add your main, supplementary figure, and table legends to the main manuscript text after the references section; remove the separate uploaded file containing the supplementary figure legends.
-Please add ORCID ID for corresponding (and secondary corresponding) author--you should have received instructions on how to do so.
-Please add a Category for your manuscript in our system.
-Please add the X and Bluesky handles of your host institute/organisation, as well as your own, and/or one of the authors, in our system.
-Please be sure that the authorship listing and order are correct and match between the system and the manuscript file.
-It is recommended to exclude figures from the manuscript text and upload them only separately.
-Please add an Author Contributions section to your main manuscript text.
-The contributions selected for Guoying Huang and Siyi Lin do not qualify them for authorship. Please either update the contributions in our system and in the Author Contributions section of the manuscript, or let us know if the authors need to be removed (and added eventually to the acknowledgment section).
-Please add a Conflict of Interest statement to your main manuscript text.
-Please add callouts for Figures S1A, B and S3A, B to your main manuscript text.
-Please be sure that the authorship listing and order is correct

We encourage our authors to provide original source data, particularly uncropped/-processed electrophoretic blots and spreadsheets for the main figures of the manuscript. If you would like to add source data, we would welcome one PDF/Excel-file per figure for this information. These files will be linked as supplementary "Source Data" files.

It is Life Science Alliance policy that if requested, original data images must be made available to the editors. Failure to provide original images upon request will result in unavoidable delays in publication. Please ensure that you have access to all original data images prior to final submission.

If you are planning a press release on your work, please inform us immediately to allow informing our production team and scheduling a release date.

We welcome submissions of potential cover images for the issue of LSA in which your work would appear. If you have high quality images associated with this work, please feel free to email these, with a caption, to the journal office.

LSA encourages authors to provide a 30-60 second video where the study is briefly explained. These videos will be appear embedded with the manuscript online at Life Science Alliance, and on social media to promote the published paper and authors (for examples, see https://docs.google.com/document/d/1-UWCfbE4pGcDdcgzcmiuJl2XMBJnxKYeqRvLLrLSo8s/edit?usp=sharing). Corresponding or first-authors are welcome to submit the video. Please submit only one video per manuscript. The video can be emailed to contact@life-science-alliance.org

FINAL FILES:

To upload the final version of your manuscript, please log in to your account: https://lsa.msubmit.net/cgi-bin/main.plex
You will be guided to complete the submission of your revised manuscript and to fill in all necessary information. Please get in touch in case you do not know or remember your login name.

The following items are required for acceptance.

-- An editable version of the final text (.DOC or .DOCX) is needed for copyediting (no PDFs).

-- High-resolution figure, supplementary figure and video files uploaded as individual files: See our detailed guidelines for preparing your production-ready images, https://www.life-science-alliance.org/authors

-- Summary blurb (enter in submission system): A short text summarizing in a single sentence the study (max. 200 characters including spaces). This text is used in conjunction with the titles of papers, hence should be informative and complementary to the title. It should describe the context and significance of the findings for a general readership; it should be written in the present tense and refer to the work in the third person. Author names should not be mentioned.

The license to publish form must be signed before your manuscript can be sent to production. A link to the license to publish form will be available to the corresponding author only. Please take a moment to check your funder requirements.

Reviews, decision letters, and point-by-point responses associated with peer-review at Life Science Alliance will be published, alongside the manuscript. If you do want to opt out of having the reviewer reports and your point-by-point responses displayed, please let us know immediately.

Thank you for your attention to these final processing requirements. Please revise and format the manuscript and upload materials as soon as you are able.

Thank you for this interesting contribution to the literature. We look forward to publishing your paper in Life Science Alliance.

Sincerely,

Sarita Hebbar, PhD
Scientific Editor
Life Science Alliance
http://www.lsajournal.org

Reviewer #1 (Comments to the Authors (Required)):

This revised manuscript has incorporated some of the suggested improvements, including JAG1 restoration experiment and promoter-activity testing. Accordingly, the report is improved in rigor. The key limitations are also acknowledged.

July 3, 2026

RE: Life Science Alliance Manuscript #LSA-2025-03597-TRR

Prof. Guoying Huang
Children's Hospital of Fudan University
399 Wanyuan Road
Shanghai 201102
China

Dear Dr. Huang,

Thank you for submitting your Research Article entitled "A cardiac-related, promoter-proximal, regulatory element shapes chromatin and JAG1 transcription". It is a pleasure to let you know that your manuscript is now accepted for publication in Life Science Alliance. Congratulations on this interesting work.

The final published version of your manuscript will be deposited by us to PubMed Central upon publication.

Your manuscript will now progress through copyediting and proofing. It is journal policy that authors provide original data upon request.

Your article will publish open access upon publication under a CC-BY license.

Reviews, decision letters, and point-by-point responses associated with peer-review at Life Science Alliance will be published, alongside the manuscript. If you do want to opt out of having the reviewer reports and your point-by-point responses displayed, please let us know immediately.

IMPORTANT: If you will be unreachable at any time, please provide us with the email address of an alternate author. Failure to respond to routine queries may lead to unavoidable delays in publication.

Scheduling details will be available from our production department. You will receive proofs shortly before the publication date. Only essential corrections can be made at the proof stage so if there are any minor final changes you wish to make to the manuscript, please let the journal office know now.

DISTRIBUTION OF MATERIALS:
Authors are required to distribute freely any materials used in experiments published in Life Science Alliance. Authors are encouraged to deposit materials used in their studies to the appropriate repositories for distribution to researchers.

You can contact the journal office with any questions, contact@life-science-alliance.org

Again, congratulations on a very nice paper. I hope you found the review process to be constructive and are pleased with how the manuscript was handled editorially. We look forward to future exciting submissions from your lab.

Sincerely,

Sarita Hebbar, PhD
Scientific Editor
Life Science Alliance
http://www.lsajournal.org
